# The necessity of a holistic approach when managing marine mammal–fisheries interactions: Environment and fisheries impact are stronger than seal predation

**DOI:** 10.1007/s13280-018-1131-y

**Published:** 2018-12-08

**Authors:** David Costalago, Barbara Bauer, Maciej T. Tomczak, Karl Lundström, Monika Winder

**Affiliations:** 1 Department of Ecology, Environment and Plant Sciences, Stockholm University, Campus Frescati, Svante Arrhenius väg 20 F, 106 91 Stockholm, Sweden; 2 Institute for the Oceans and Fisheries, University of British Columbia, UBC-AERL, 2202 Main Mall, Vancouver, BC V6T 1Z4 Canada; 3 Baltic Sea Centre, Stockholm University, Campus Frescati, Svante Arrhenius väg 20 F, 106 91 Stockholm, Sweden; 4 Department of Aquatic Resources, Swedish University of Agricultural Sciences (SLU), Turistgatan 5, 45330 Lysekil, Sweden

**Keywords:** Atlantic cod, Atlantic herring, EwE, Fisheries management, Marine mammals, Sprat

## Abstract

Seal populations are recovering in many regions around the world and, consequently, they are increasingly interacting with fisheries. We used an Ecopath with Ecosim model for the offshore Central Baltic Sea to investigate the interactions between the changes in fish stocks and grey seal (*Halichoerus grypus*) population under different fishing and environmental scenarios for the twenty-first century. The assumed climate, eutrophication and cod (*Gadus morhua*) fisheries scenarios modified seal predation impacts on fish. Fish biomass and catches are more affected by fishing mortality and the environment than by seal predation. Our results highlight that the impacts of the increasing seal population on lower trophic levels are complex; thus, we emphasize the need to consider a range of possible ecosystem contexts when evaluating potential impacts of top predators. Finally, we suggest that an increasing seal population is not likely to hinder the preservation of the main Baltic fish stocks.

**Electronic supplementary material:**

The online version of this article (10.1007/s13280-018-1131-y) contains supplementary material, which is available to authorized users.

## Introduction

During the second half of the twentieth century, conservation efforts led to the improvement of the status of numerous marine mammal populations worldwide (Lotze et al. 2011; Magera et al. 2013; Chasco et al. 2017). Many marine mammal species feed mostly on fish, which consequently leads to a polarized discussion regarding the potential effects of these animals on fish catches. For example, 80% of all seal species worldwide, including phocids (true seals), otarids (eared seals) and walrus, have been recorded to have some form of negative effect on fishing or fish farm operations (Wickens 1995; Trzcinski et al. 2006). However, in many cases, the conflicts between piscivorous predators and fishery arise from poor understanding of the system’s complexity of predator-prey interactions and lack of consideration of other pressures affecting the food web.

Many studies quantify the losses to fishery without taking into account that (1) not every fish is caught, even if predators are lacking (Yodzis 2001; Heikinheimo et al. 2016), and (2) there might be several confounding factors shaping the seals’ potential for predation (e.g. climate change, competitors and diseases (O’Boyle and Sinclair 2012; Morissette and Brodie 2014). Thus, estimating the seals’ trophic impact on their prey populations is challenging but important to evaluate the extent of the top-down control by seals. In addition, marine ecosystems are being rapidly altered by climate change, fishing activities and eutrophication, among other factors. It is thus necessary to develop tools that allow us to understand and to predict the effects of a changing environment on marine mammals and the interactions between their populations and fish stocks. This understanding can inform the debate on the conservation and management of marine mammals and fisheries and alleviate conflicts.

In the northern hemisphere, the most abundant pinniped species are grey seal *Halichoerus grypus,* harbour seal *Phoca vitulina*, harp seal *Pagophilus groenlandicus* and ringed seal *Pusa hispida*, although harp and ringed seals are typically arctic species (Perrin et al. 2009). In Europe, seals have been reported to interact with the fisheries in some of the most important fishing grounds, e.g. the North Sea (Furness 2006), the Barents Sea (Bogstad et al. 2015) and the Baltic Sea (Varjopuro 2011; Gårdmark et al. 2012). Thus, there is a growing concern regarding the impact that grey seals might have on some of the most important fish stocks in the region, e.g. Eastern Baltic cod (*Gadus morhua*) and Atlantic herring (*Clupea harengus*). For the Baltic Sea, the exponential increase of the grey seal population since the 1980s raised concerns in the fisheries sector (Lundström et al. 2010; Varjopuro 2011). This region experienced, in addition, vast impacts of climate change and eutrophication (MacKenzie et al. 2012; Niiranen et al. 2013; Meier et al. 2014; Elmgren et al. 2015), which make this region a suitable study case to estimate the extent to which the increasing number of seals interact with fisheries under different environmental scenarios.

**Fig. 1:**
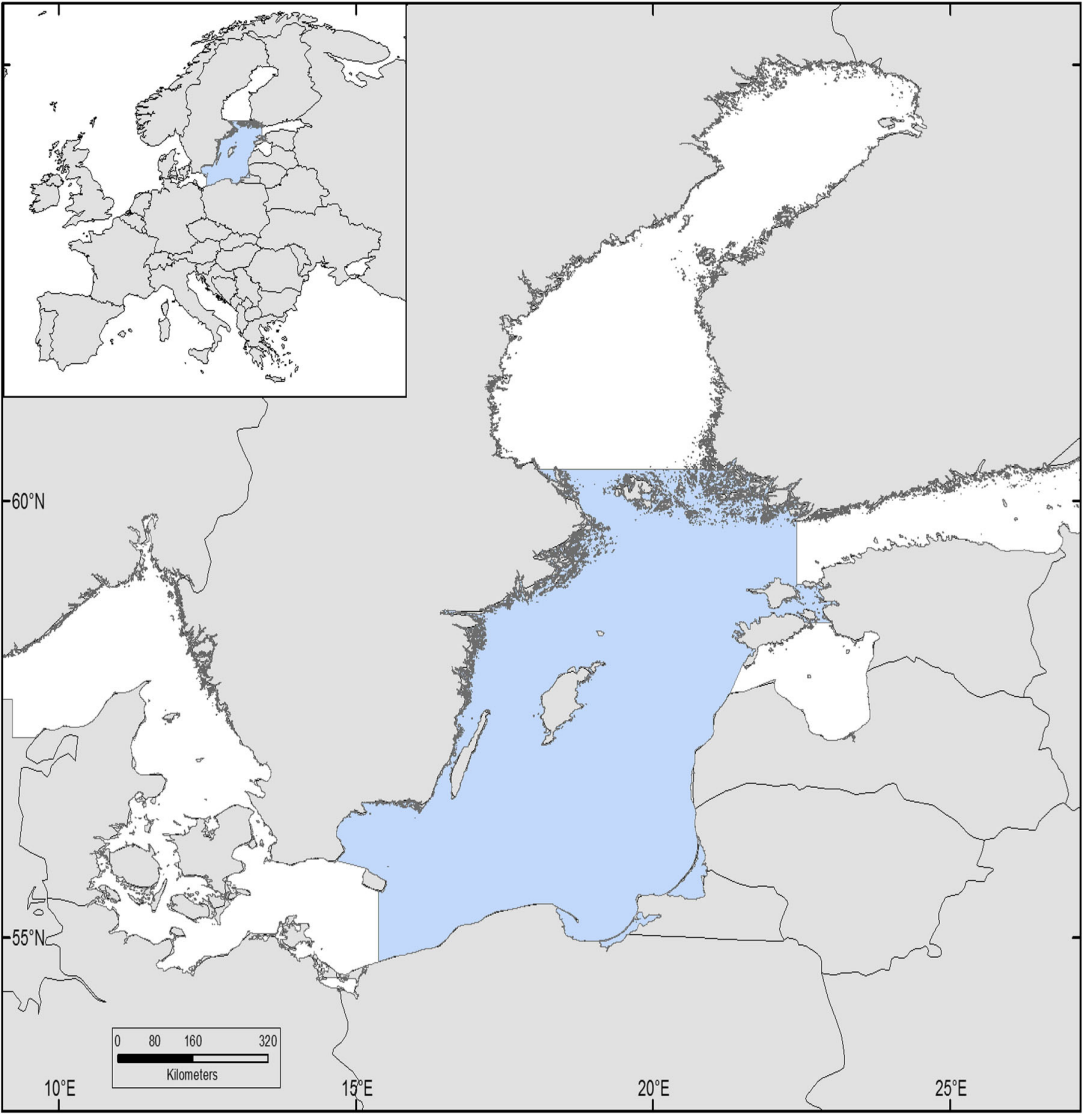
The Baltic Proper study area (blue) includes the ICES subdivisions 25–27, 28–2 and 29

Here, we used the Baltic Proper (Fig. 1) as a model to investigate the role of grey seals in a system highly affected by multiple human pressures. We aimed at quantifying the contribution of grey seal predation on the most economically important fish stocks in the Baltic Sea (i.e. Eastern Baltic cod, Baltic herring and sprat (*Sprattus sprattus*)) under different future environmental conditions. Ultimately, we aim at providing new insights for management and conservation from an ecosystem perspective, and to aid at resolving conflicts where fisheries and seals interact.

## Materials and Methods

### Study area

The Baltic Sea is one of the world’s largest brackish water bodies. This area hosts four resident species of marine mammals, grey seal being the most abundant of them. The Baltic Proper area covered in the model (Fig. 1) extends approximately 2.4 × 10^5^ km^2^. Most Baltic grey seal individuals concentrate in the Baltic Proper (Härkönen et al. 2013) and the largest stocks of Eastern Baltic cod, Baltic herring and Baltic sprat are also found in that area (ICES 2017b). Landings of herring from the pelagic fisheries reached a peak in the mid-1970s. In the late 1980s, a decline in Baltic cod abundances led to a decrease in landings (Casini et al. 2008). The Eastern Baltic cod stock in the Baltic Proper showed weak signs of recovery in the beginning of the twenty-first century (Gårdmark et al. 2015; Raid et al. 2016), whereas the central Baltic herring stock biomass, although currently increasing and above safe biological limits, is still about half the size it was in the 1970s. However, it is uncertain whether these increases will continue (Svedäng and Hornborg 2015). Sprat biomass increased rapidly in the late 1980s–early 1990s, but before the start of the twenty-first century, it decreased to values similar to those in 1970s (Eero 2012).

### Ecopath with Ecosim model parameterization

Ecopath with Ecosim (EwE) is a widely used food-web modelling approach (Christensen and Walters 2004). It consists of Ecopath, a mass-balanced, static model describing trophic flows among functional groups in one year (in this case 1974), and Ecosim, a dynamic simulation model. To estimate the magnitude of the trophic fluxes, an Ecopath model requires input values for parameters such as biomass, diet composition, consumption, fisheries catches and production rates for each functional group (Table S1–S4).

In order to simulate the trophic effects of grey seals on the fish populations as realistically as possible, the existing Baltic Proper Ecopath model (Tomczak et al. 2012, 2013) was updated (Appendix S1). This EwE model was built for the open sea area of the Baltic Proper to describe its food-web dynamics between 1974 and 2006 in order to understand the changes in energy flow and the observed regime shift in the Baltic Sea ecosystem. We assumed that all functional group biomasses were in equilibrium in 1974 (i.e. the ‘biomass accumulation’ parameter was set to zero for all groups in the Ecopath model) and that there was no significant migration to or from areas outside of the Baltic Proper.

Diet compositions of all functional groups in the model except the seals were kept as described by Tomczak et al. (2012). Briefly, adult and small cod fed primarily on juvenile stages of herring and sprat. A certain degree of cannibalism (6% of total diet composition) and consumption of prey from outside the system (‘import’, 8% of total diet composition) was also accounted for in adult cod. Juvenile cod fed mostly on macrozoobenthos and mysids, and larval cod fed on copepods. Juvenile and adult herring fed mainly on copepods and mysids, and juvenile and adult sprat fed on copepods.

**Fig. 2:**
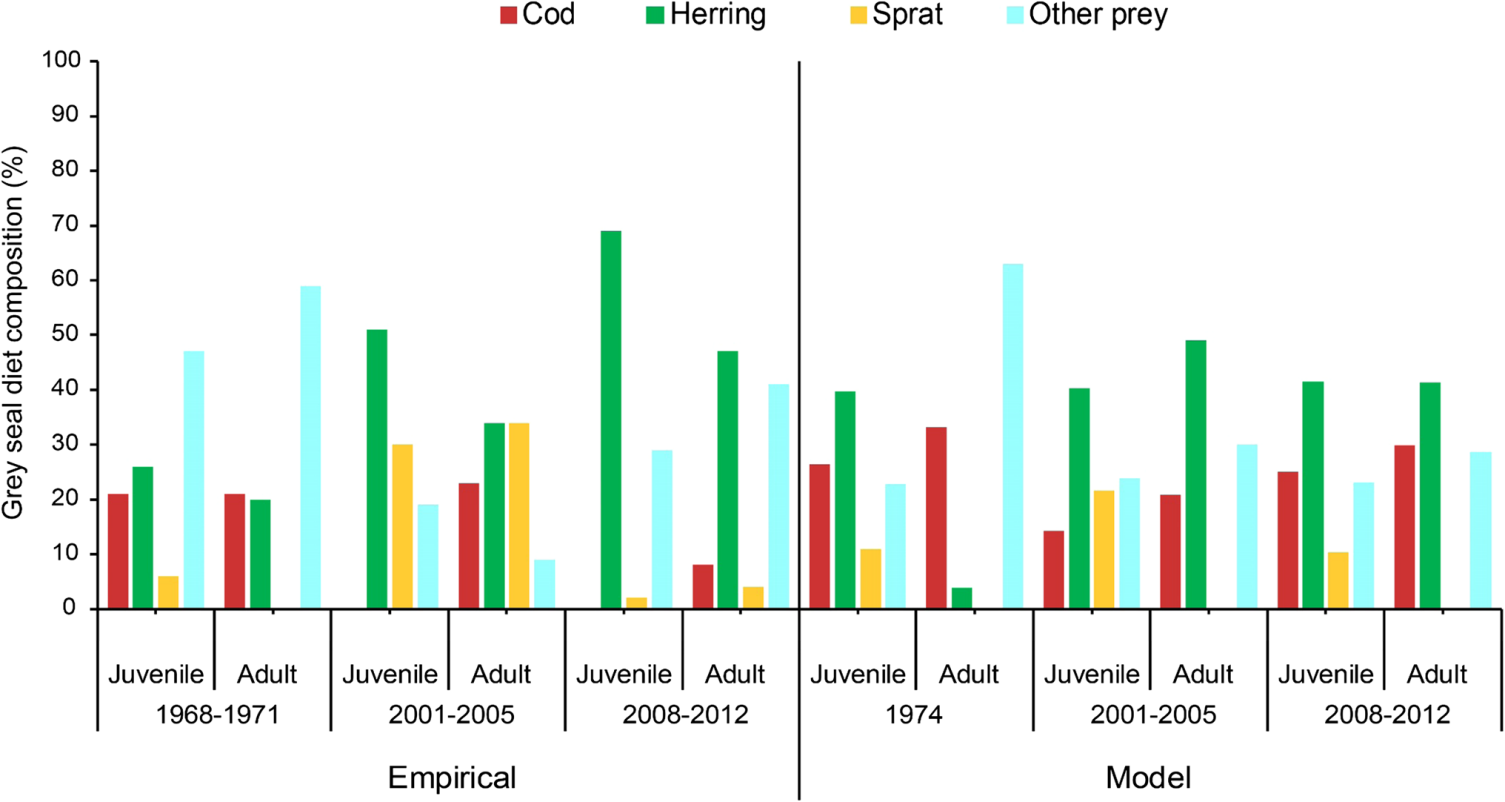
Composition, in % biomass, of juvenile and adult grey seal diets in the Baltic Proper according to digestive tract content analyses (left panel) from the periods 1968–1971 (Söderberg 1975), 2001–2005 (Lundström et al. 2010) and 2008–2012 (samples from hunted seals analysed for this study following the methods in Lundström et al. (2010)), and our model grey seal diet outputs (right panel) for 1974, 2001–2005 and 2008–2012

Individual seal consumption was considered to be 6 kg day^−1^ for juvenile seals and 6.9 kg day^−1^ for adults (6 kg day^−1^ for females and 7.8 kg day^−1^ for males), as estimated by Lundström (2012). Grey seal diets in the Baltic Proper in the periods 1968–1971, 2001–2005 and 2008–2012 were obtained from hunted seals from Söderberg (1975), Lundström et al. (2010) and samples from hunted seals analysed for this study following the methods in Lundström et al. (2010), respectively (Fig. 2). The diet composition in the period 1968–1971 was used as a starting point to parameterize the grey seal diet composition in Ecopath but was modified during the model balancing procedure (see next section).

The Ecosim model has the balanced Ecopath as initial state and simulates how the ecosystem changes compared to that state following one or more forcing factors. Biomass changes in non-age structured groups (all except seals and fish) are modelled as differential equations, based on the Ecosim master equation where biomass change is a function of prey consumed minus losses by predation, fisheries and other mortality. Fish biomasses are modelled by a monthly difference equation system, accounting for changes in population age and size structure in each time step. The model assumes a von Bertalanffy growth curve and weight-dependent fecundity, where weight depends on the amount of prey consumed (Walters et al. 2010). Seal biomass changes are either used as scenario forcing (seal0, seal1 scenarios, Table 2) or modelled similarly to that of fish (seal50, Table 2). Predator diet compositions depend on prey abundances and on the predator preferences (‘electivities’ in EwE nomenclature) determined in Ecopath (Appendix S1, Fig. S1). After the Ecopath model is balanced, there are still a few additional parameters required by Ecosim (Appendix S2; regarding setting the vulnerability multipliers see text below).

### Model balancing and validation

To ensure that the Ecopath model estimates were balanced and realistic, we used the PREBAL procedure (Link 2010). Additionally, model estimates were analysed by comparing the observed and simulated biomass and catches. We also compared the empirical seal diet data of the three periods, i.e. 1968–1971, 2001–2005 and 2008–2012 with the Ecopath output seal diet obtained from the ‘Ecopath from Ecosim’ feature for the years 1974, 2001–2005 and 2008–2012, respectively, to assess whether the diet compositions estimated by the model were realistic.

Subsequently, the Ecosim module was used to create time-dynamic simulations of the food web in response to historical (1974–2015) fishing, environmental and seal biomass forcing. Fishing mortality data for herring and sprat were used as forcing in the model and were obtained from ICES (ICES 2017a) (Fig. S2). The impact of environmental factors (i.e. primary production (PP), sea temperature, salinity, cod reproductive volume and hypoxic area) on the functional groups was modelled using forcing functions (Table 1, Fig. S2, see also Niiranen et al. (2013) and Meier et al. (2014) for details on forcing variables). The environmental variables were chosen based on existing literature on the most important environmental drivers affecting the Baltic Sea food web (Niiranen et al., 2013).

**Table 1:** Forcing variables used in the Ecosim model and their respective target group in the EwE model. All environmental forcing variables were applied as anomalies from the Ecopath base year (1974) for the period 1974–2098

Forcing variable	Target group
Sea surface (0–10 m) temperature in August; proxy of sprat egg production	Juvenile sprat
Upper water column (0–50 m) temperature in spring	*Acartia* spp., *Temora longicornis*
Lower water column (80–100 m) salinity, annual average	*Pseudocalanus acuspes*
Phytoplankton production per biomass (P/B), annual	Phytoplankton
Hypoxic area, annual average	Macrozoobenthos, mysids
Cod reproductive volume (volume of water with salinity > 11 psu and deep water oxygen concentration > 2 mg l^−1^), annual average; proxy of egg production	Cod larva
Herring recruitment, annual average biomass of age 1 class, proxy of egg production	Juvenile herring
Seal biomass, annual average	Juvenile seal, Adult seal
Fishing rate (F), defined as yield per biomass (Y/B)	Ad. Cod, Small cod, Ad. Herring, Juv. Herring, Ad. Sprat, Juv. Sprat

Environmental forcing variables were based on projections from the model BAltic sea Long-Term large-Scale Eutrophication Model (BALTSEM; see (Savchuk et al. 2012a)); fishing mortality forcing and seal biomass forcing were based on observations in the historical period. See Tomczak et al. (2012) and Niiranen et al. (2013) for further details and the data sources of the forcing variables. Forcing function time series (1974–2015) are plotted in Fig. S2

The Ecosim model was calibrated using an automated stepwise fitting procedure that searched for vulnerability multiplier parameters that maximized statistical fit to observed time series (Scott et al. 2015). We used observation data on relative biomasses and catches (1974–2015), obtained from ICES (Tomczak et al. 2012; ICES 2017a) for model calibration. Vulnerability multipliers are also called ‘flow control parameters’ and are used to limit the biomass flow between a predator and its prey (for more details see Appendix S2).

In addition to adjusting vulnerability multiplier parameters via stepwise fitting, we also adjusted the diet matrix. In the diet matrix, we reduced the percentage of the import diet by accounting a proportional fraction of it into the different prey groups. The final diet matrix and the ‘Electivity’ are shown in Table S4 and Fig. S1, respectively. To allow grey seals to switch diet in the model (i.e. to start preferentially consuming a prey that has become more abundant), we assigned the highest possible value (2) to the ‘Switching Power’ parameter in the Group Info interface for both juvenile and adult seals.

### Scenario simulations (2016–2098)

During the scenario simulations, we varied the same forcing variables (Table 1, Fig. S2) used to force the model in the historical period. Table 2 details the seal abundance, *F*_cod_ and environmental scenarios used in the future projections (2016–2098). In the seal0 and seal1 scenarios, we used seals as ‘forcing’, i.e. set their biomasses to certain values a priori, excluding bottom-up effects affecting their biomass. In seal50, we forced the seal biomass to grow exponentially, following the current growth trend, until a maximum seal population size of 50 times the initial biomass from 1974, which is past the number of seals that there were in the Baltic Sea in the beginning of the twentieth century [nearly 100 000 individuals (Harding and Härkönen 1999)]. This is followed by a stabilization of seal biomass around 2 040 of 0.07 t km^−2^ or 140 000 individuals (Fig. S3). The Multi-sim functionality of Ecosim was used to simulate the dynamics of the groups from 2016 to 2098 for each of the future scenarios. Linear models were fitted to the log-transformed model outputs for the period 2016–2098 to investigate how the environmental, *F*_cod_ and seal abundance scenarios affect the mean annual biomass, catches, seal consumption and fish predation mortality by seals of adult cod, herring and sprat in each scenario combination (Fig. S4). No carrying capacity limits were set for any of the functional groups.

**Table 2:** Description of scenarios used in the projections (2016–2098) in the food-web EwE model. Fishing mortalities of sprat and herring were kept constant during the scenarios at their respective 2015 levels (i.e. *F*_herring_ = 0.11 and *F*_sprat_ = 0.21). The environmental scenarios were named Env0, Env1 and Env2, indicating increasing anthropogenic pressures on the Baltic Sea

Seal abundance scenarios	
seal0	Total removal of grey seals after 2015.
seal1	Constant at the 2015 abundance (i.e. around 27 000 individuals).
seal50	An exponential growth following the current growth trend was set to a maximum seal population size of 50 times the initial biomass from 1974 in the projections (i.e. from 1 750 adult seals in 1974 to 85 000 adult seals in 2 098), which ultimately yields a total seal biomass in the Baltic Proper similar to the estimated population size for the entire Baltic Sea in the beginning of the twentieth century [i.e. 80 000–100 000 individuals (Harding and Härkönen 1999)].
Fishing scenarios of eastern Baltic cod fishing mortality (*F*_cod_)	
Fcod0.3	Fishing mortality of Eastern Baltic cod (*F*_cod_) according to the European Union recovery plan (Regulation 2007), i.e. *F*_cod_ = 0.3.
Fcod1	*F*_cod_ = 1, corresponding to the limit reference point for *F*_cod_ that is expected to drive the stock to the biomass limit. This value also coincides with the average *F*_cod_ between 1974 and 2006 (ICES 2007), before the 2007 Management Plan was established (EC 2007).
Environmental scenarios^a^	
Env0	
Climate scenario	No change in average air temperature, precipitation and wind relative to year 2015 conditions.
Nutrient load scenario	Reduction of riverine nutrient discharges following Baltic Marine Environment Protection Commission (HELCOM) Baltic Sea Action Plan (BSAP) (HELCOM 2007).
Env1	
Climate scenario	Warming scenario according to global climate model ECHAM5; + 2.8 °C mean temperature and + 12% precipitation changes over the Baltic Sea region for 2070–2099 relative to 1969–1998 (Meier et al. 2012).
Nutrient load scenario	Present (2015) nutrient concentration in rivers (see Savchuk et al. (2012a)).
Env2	
Climate scenario	Warming scenario according to global climate model HadCM3; + 3.8 °C mean temperature and + 18% precipitation changes over the Baltic Sea region for 2070–2099 relative to 1969–1998 (Meier et al. 2012).
Nutrient load scenario	Business-as-usual for nutrient concentrations in rivers assuming an exponential growth of fertilizers use in agriculture in all Baltic Sea countries following HELCOM (2007).

^a^Environmental scenarios (Env) were produced by combining regionally downscaled global climate scenarios from the Intergovernmental Panel on Climate Change (IPCC) with nutrient load scenarios generated by the biogeochemical model BAltic sea Long-Term large-Scale Eutrophication Model (BALTSEM; see (Savchuk et al. 2012a)). For further details about the regionally downscaled global climate scenarios, see Meier et al. (2012, 2014) and Niiranen et al. (2013), and for the nutrient load scenarios see (Savchuk et al. 2012b). We assumed Env0, Env1 and Env2 to be the best-case, intermediate and worst-case environmental scenarios, respectively, for the Baltic Sea

### Mixed Trophic Impact (MTI)

Ecopath uses the Network Analysis routine called Mixed Trophic Impact (MTI) to estimate the direct and indirect effects (positive or negative) that a change in the biomass of one functional group might have on another group’s biomass (Appendix S3). To obtain the MTI for all modelled years (1974–2098), we used the ‘Ecopath model from Ecosim’ tool. This tool generates a new Ecopath model for each of the years projected in Ecosim. We present here the effect (measured by MTI) of the adult seal group and of the cod trawl fishery on the fish, adult seal and fisheries groups in the Env1 + seal50 + *F*_cod_ = 0.3 scenario combination. The Env1 scenario was selected for the MTI analysis as this was an ‘intermediate’ scenario in terms of environmental impacts. The seal biomass and cod fishing mortality scenarios seal50 and *F*_cod_ = 0.3 were chosen because we wanted to assess the cod mortality under the strongest possible impact of seals while keeping *F*_cod_ within the former European Union Council recovery plan (EC 2007).

In addition, given the relatively high proportion of prey outside the system in our model, we tested whether the MTI of seals on the prey would be different when eliminating the ‘import’ diet for seals and redistributing those values proportionally into the different prey groups. We tested this in the year 2094 and at seal50 to make sure seal abundance was at or near its potential peak.

## Results

### Model calibration and validation

Our Ecopath model followed PREBAL ecological rules of thumb as described by Link (2010) (Fig. S5). Among all vulnerability multipliers, those that regulated the interactions of cod with its prey influenced model fit the most. The best fit for seal biomass was obtained when increasing the seals Switching Power to 2 and their prey vulnerability multipliers to ≫ 2 (Appendix S2). Therefore, we assumed high vulnerability for the seal prey groups (i.e. juvenile and adult sprat, juvenile and adult herring, and juvenile, small and adult cod).

Grey seal diet projections for the years 1974, 2004 and 2010 were relatively similar to the empirical data for the periods 1968–1971, 2001–2005 and 2008–2012, respectively (Fig. 2). In the period 1968–1971, cod was a very important part of the diet for both juvenile and adult seals (21%) and herring was of particular relevance in the juvenile seal diet (26%), while sprat was of relative little importance compared to the other periods, both in our model (1974) and in the empirical data. During the other periods (2001–2005 and 2008–2012), both the model and the empirical data coincided in a major contribution of herring in the diet of seals. Other prey (e.g. salmon, eel, perch, flatfish, whitefish) were generally more important than cod, sprat or herring in the seal diet according to both the model and the observations in the first period (Fig. 2). In general, cod seemed to be more important in the diet of seals according to the model results than according to the empirical data (Fig. 2).

### Scenario simulations

Under the seal50 scenario, the grey seal biomass was set to grow exponentially a peak of about ~ 141 000 individuals, including both juvenile (56 640 individuals) and adult (84 840 individuals) seals, and then the seal biomass was forced to level off after the year 2039.

#### Adult fish biomass

**Fig. 3:**
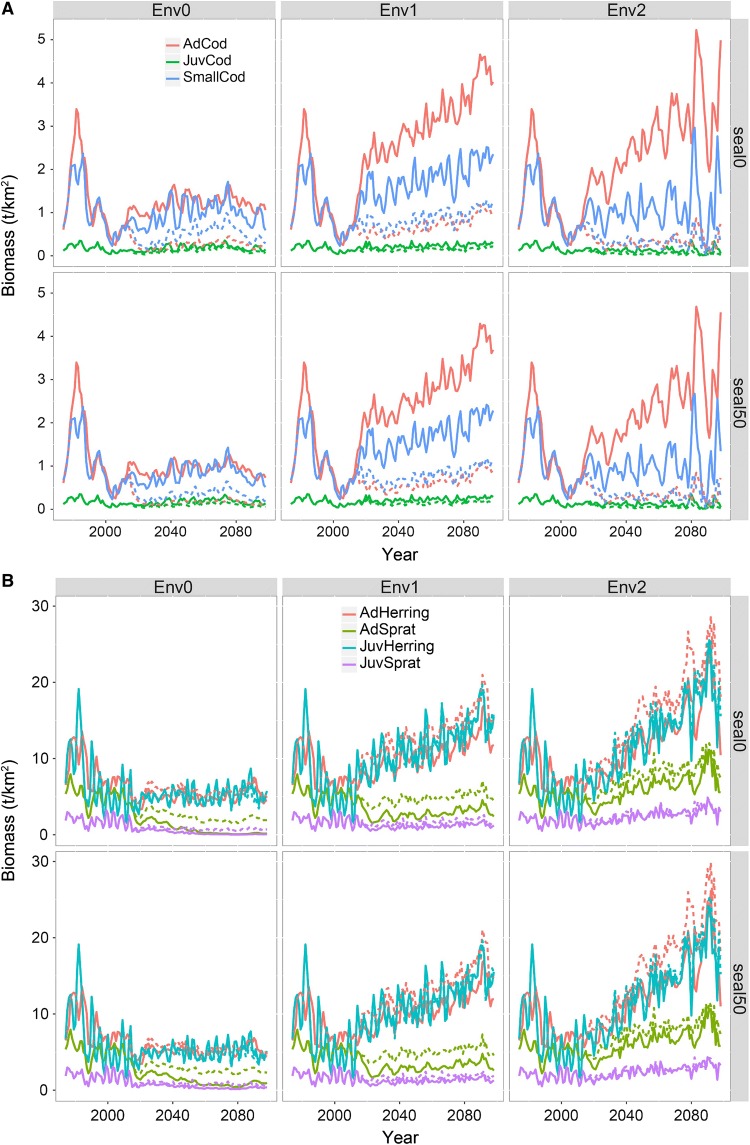
Cod (**a**), and herring and sprat (**b**) biomasses from 1974 to 2098 under different environmental (columns: Env0, Env1 and Env2), seal abundances (rows: seal0 and seal50) and cod fishing mortality (*F*_cod_0.3 as continuous lines and *F*_cod_1 as dotted lines) scenarios. *AdCod* adult cod, *JuvCod* juvenile cod, *SmallCod* small cod, *AdHerring* adult herring, *AdSprat* adult sprat, *JuvHerring* juvenile herring, *JuvSprat* juvenile sprat

The projections time series from 1974 to 2098 show that adult and small cod biomasses under the Env0 scenario remain rather stable from 2015 regardless of *F*_cod_ and of seal abundance (Fig. 3a). At Env1 and at Env2, and under *F*_cod_ = 0.3, adult and small cod biomasses increase constantly over time under both seal0 and seal50 scenarios, with a steeper slope at Env1 than at Env2 (Fig. 3a). Overall, adult cod biomass was between 1.9 and 7.9 times lower under *F*_cod_ = 1 than under *F*_cod_ = 0.3 (Table S5). When *F*_cod_ = 0.3, adult cod biomass was 13% lower under seal50 than under seal0, whereas when *F*_cod_ = 1 cod biomass was less than 6% lower under seal50 than under seal0 (Table S5). Differences in adult cod biomass between *F*_cod_ = 0.3 and *F*_cod_ = 1 were significant (*p* < 0.05) in all environment scenarios. At *F*_cod_ = 0.3, regardless of the seal abundance, the Env1 and Env2 environmental scenarios yielded higher adult cod, herring and sprat biomass than the Env0 scenario (Fig. S6A). At *F*_cod_ = 1, regardless of the seal abundance, cod biomass reached the highest values in the Env1 scenario and the lowest values in the Env2 scenario, whereas herring and sprat biomass followed the same pattern as under *F*_cod_ = 0.3 (Fig. S6A).

The adult and juvenile herring biomasses in Env0 scenario remain stable from 2015 to 2098 regardless of *F*_cod_ and of seal abundance (Fig. 3b). At Env1 and at Env2, herring biomass increases constantly over time under both seal0 and seal50 scenarios, but with a steeper slope at Env2 than at Env1 (Fig. 3b). Adult herring biomass was 13–23% lower under *F*_cod_ = 0.3 than under *F*_cod_ = 1 (Table S5), with significant differences between environment scenarios (e.g. around 2.6 higher under Env2 than under Env0) and between *F*_cod_ scenarios, but not between seal scenarios (Table S5, Fig. S6A).

The adult and juvenile sprat biomasses in Env0 scenario decrease slightly during the simulated period regardless of *F*_cod_ and of seal abundance (Fig. 3b). At Env1 and at Env2, sprat biomass increases constantly over time under both seal0 and seal50 scenarios, but with a steeper slope at Env2 than at Env1 (Fig. 3b). The highest sprat biomass of all environmental scenarios was found under Env2, and the lowest biomass under Env0 (Table S5, Fig. S6A): in Env2 sprat biomass was 5, 7 or 8 times higher than in Env0, under seal50, seal1 and seal0, respectively, when *F*_cod_ = 0.3. However, under *F*_cod_ = 1 sprat biomasses were consistently higher than under *F*_cod_ = 0.3 and the differences in sprat biomass between Env scenarios were smaller (Table S5), suggesting that there is an important indirect effect of *F*_cod_ on sprat biomass.

#### Seal consumption (Q)

Juvenile and adult seal consumption (Q) of adult cod was between 16 and 93 times higher under *F*_cod_ = 0.3 than under *F*_cod_ = 1, depending on the environmental and seal abundance scenarios (Q of cod was nearly 0 when *F*_cod_ = 1). When *F*_cod_ = 0.3, Q of cod was significantly lower under seal1 than under seal50, and it was ~ 50% lower in the Env0 scenario than in the other two environmental scenarios (Table S6).

Seal consumption of adult herring was approximately 3 times higher when *F*_cod_ = 1 compared to *F*_cod_ = 0.3. Under *F*_cod_ = 0.3, Q of herring was significantly higher at seal50. Overall, Q of herring was 1.5–2 higher in the Env2 scenario than in the other two environment scenarios (Table S6). Under *F*_cod_ = 1, Q of sprat was generally 2–3 higher in the Env2 scenario than in the other two environment scenarios for both seal1 and seal50 scenarios, whereas under *F*_cod_ = 0.3 the difference in sprat biomass between Env2 and the other environmental scenarios was more than double than under *F*_cod_ = 1 (Fig. S6B) (Table S5).

#### Adult fish catch

Adult cod catches were between 1.3 and 2.4 times higher under *F*_cod_ = 0.3 than under *F*_cod_ = 1. Cod catches were significantly different between environment scenarios (Env1 > Env2 > Env0), with higher differences when *F*_cod_ = 0.3 than when *F*_cod_ = 1. Adult herring and adult sprat catches followed patterns similar to the ones described above for their biomasses (Fig. S6C, Table S7). At seal50 and *F*_cod_ = 0.3, Q of adult cod was 50–80% higher than adult cod catches (Tables S6 and S7).

#### Predation mortality

**Fig. 4:**
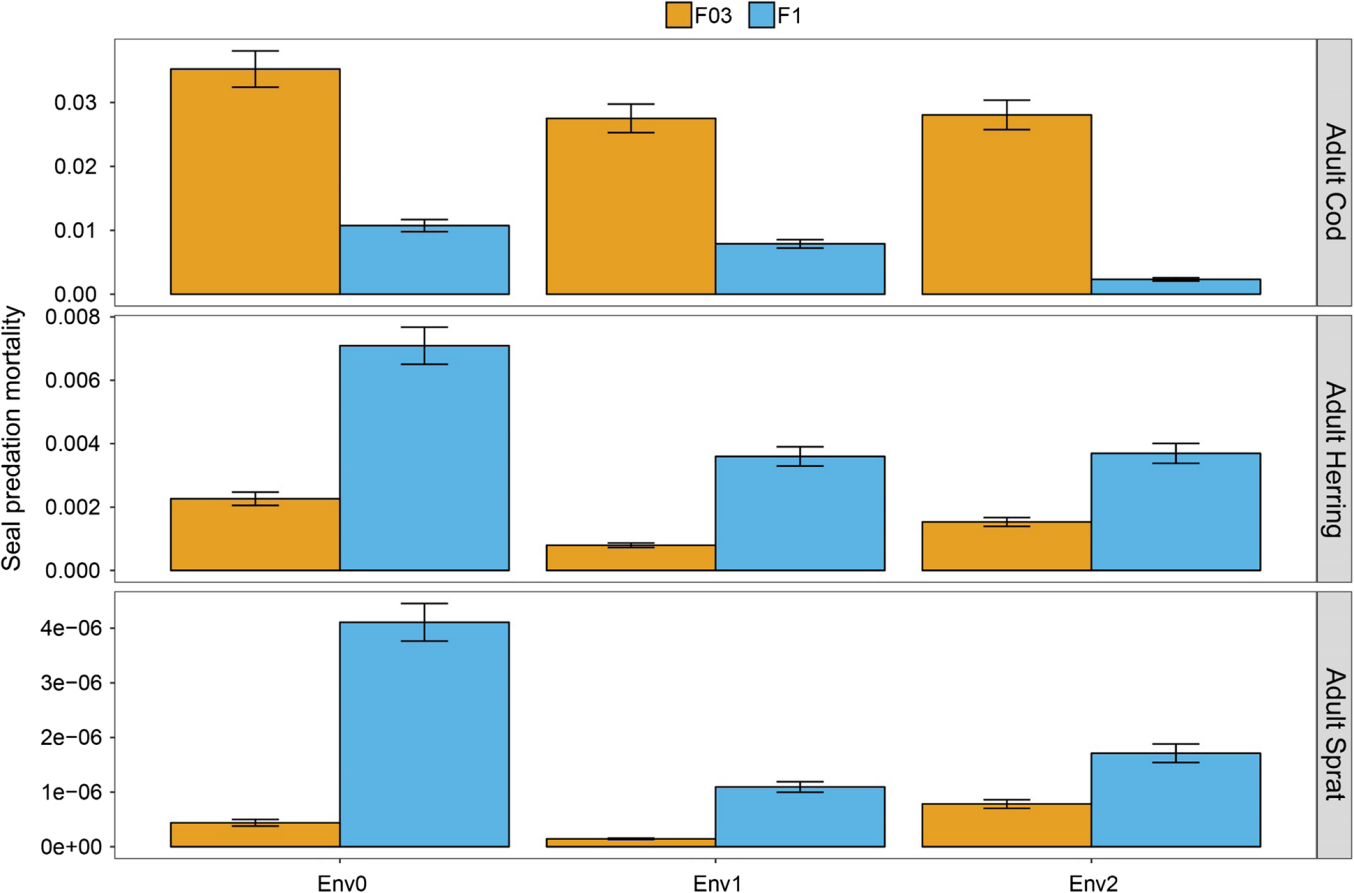
Predation mortality by seals of adult cod, herring and sprat under environmental scenario combinations of *Env0, Env1 and Env2* and cod fishing mortality scenarios *F*_cod_ = 0.3 (orange) and *F*_cod_ = 1 (blue) for the period 2015–2098. Only the seal50 scenario is represented here, as predation mortality of these prey at seal1 was near 0

Predation mortality of adult cod by seals was significantly higher (*p* < 0.05) under *F*_cod_ = 0.3 than under *F*_cod_ = 1. Within both *F*_cod_ scenarios, the predation mortality of adult cod by seals was higher under the Env0 scenario than under the other two environment scenarios (Fig. 4). For both herring and sprat, predation mortality by seals was also significantly higher under Env0, but, in opposition to cod, a much higher predation mortality by seals occurred when *F*_cod_ = 1 (Fig. 4). Only when *F*_cod_ = 0.3 and seal50, the consumption of adult cod by seal was larger than the catches.

### Mixed Trophic Impact

**Fig. 5:**
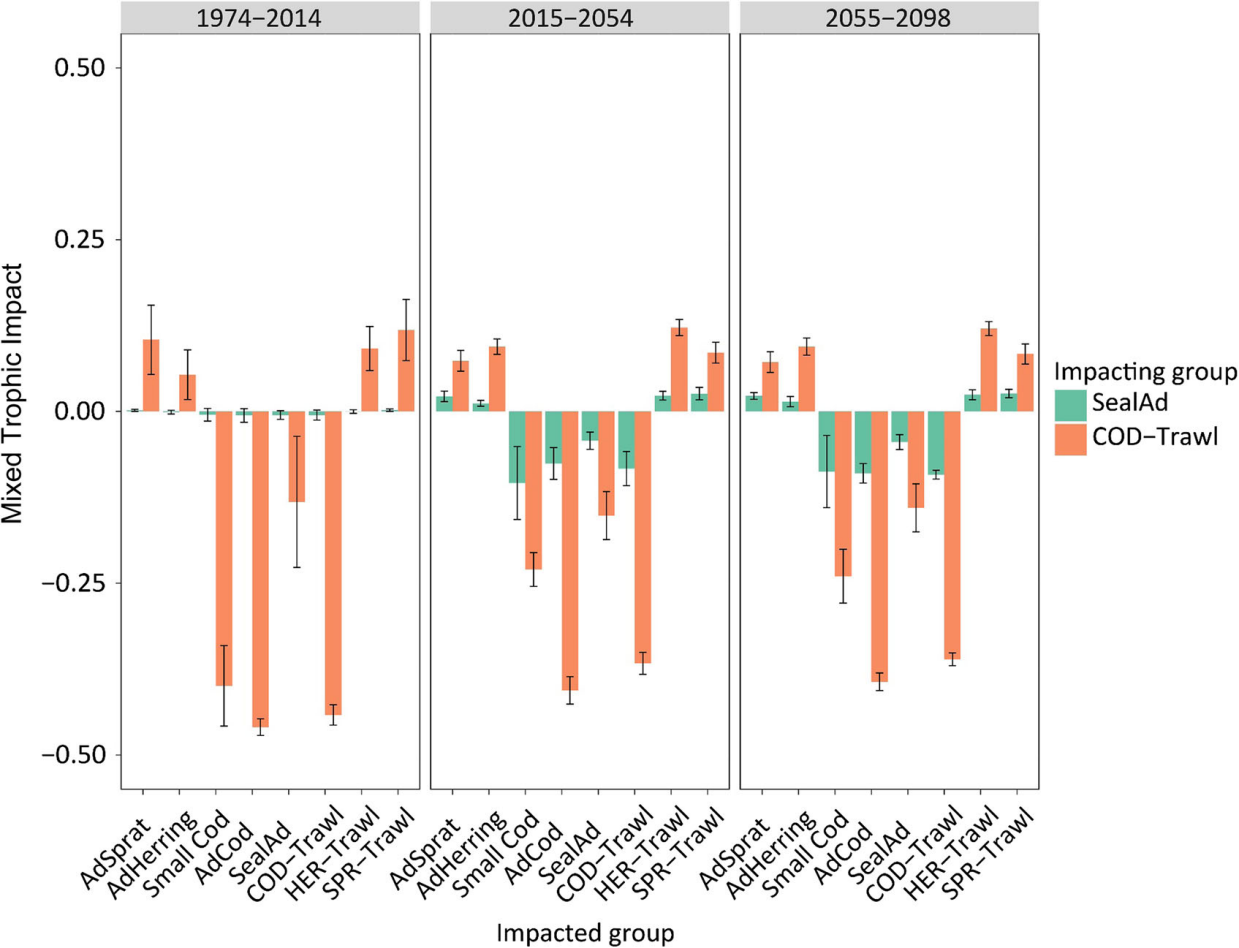
Mixed Trophic Impact (MTI) by adult seals (SealAd) and cod fishery (COD-Trawl) on the small cod, the adult fish groups and seals and on the cod, herring (HER-Trawl) and sprat (SPR-Trawl) fisheries. *AdHerring* adult herring, *AdSprat* adult sprat, *AdCod* adult cod, *SealAd* adult seal

The MTI of adult grey seal and cod fishery showed that both seals and the fishery have an overall negative impact on cod biomass (MTI_seal_ = − 0.06, MTI_codFishery_ = − 0.35) and on themselves (MTI_seal_ = − 0.03, MTI_codFishery_ = − 0.06, and MTI_seal_ = − 0.14, MTI_codFishery_ = − 0.39, by adult seal and cod fishery, respectively). From 1974–2014 to 2015–2054, the MTI of seals became more negative on cod, cod fishery and adult seal, but increased on the other fish species and fisheries. Cod fishery had an overall less negative impact during 2015–2098 than during 1974–2014, whereas the MTI of cod fishery on the other groups remained relatively unchanged over the entire time period. The MTI of adult seal and cod fishery do not vary between the 2015–2054 and 2055–2098 time periods (Fig. 5).

## Discussion

Our results showed that environment and fisheries scenarios influenced seal predation impacts on fish. Fish biomass and catches are more affected by fishing mortality and the environment than by seal predation. Therefore, this study reveals that the relationships between seal population, fish catches and fish biomass are more complex than previously stated (Wickens 1995; Trzcinski et al. 2006). Even with the relatively high adult seal consumption values used in our model (6.9 kg day^−1^) compared to previous studies (e.g. 4.3 kg day^−1^ and 3.2 kg day^−1^ (Innes et al. 1987 and Elmgren 1989, respectively)), seal abundance generally did not have substantial effects on the adult fish biomasses.

The little impact of seals on fish populations shown by our study may seem counterintuitive. However, simple energetic calculations support our results. Given consumption rates of 2.52 t fish/adult seal and 2.19 t fish/juvenile seal per year, we calculated that a total of 100 000 seals in the study area (240 000 km^2^) would consume 238 600 tons of fish per year. The model output for the seal diet composition in the period 2008–2012 showed that herring composed ∼ 41% of the diet for both juvenile and adult seals. Thus, seals consume 97 800 tons of herring per year. Based on otolith sizes, it was estimated that approximately 50% of the herring identified in the digestive tract of grey seals were adult fish consumed in the Baltic Proper (see Lundström et al. 2007 for prey size estimations). Thus, 100 000 seals would consume ∼ 49 000 tons of adult herring. This indicates that our simulation estimates of seal consumption are similar to empirical studies (Lundström 2012).

The spawning stock biomass (SSB) for herring was estimated 1 341 625 tons in ICES subdivisions 25–29 and 32 (excluding Gulf of Riga) in 2017 (ICES 2017a); therefore, the 49 000 tons of adult herring consumed by the seals represent 3.65% of the estimated herring stock size in 2017. According to our model, at the end of the simulated period (year 2098) in the Env0, Fcod0.3 and seal50 scenarios (which is the combination of scenarios yielding the lowest biomass of adult herring in 2098) adult herring biomass would be 9.15% higher than in the year 2017 (juvenile herring biomass would be 20.65% higher in 2098 than in 2017). This suggests that ∼ 100 000 seals would be eating 3.34% of herring SSB at the end of the twenty -first century. Also, predation mortality of herring (and sprat) is particularly low in the Fcod0.3 scenario in comparison to the Fcod1 scenario. This is likely because whenever cod is available (which would be the case if Fcod is low), grey seals tend to prey more on cod than on other species, as suggested by the fact that cod was as common as herring in the seal stomachs during the period 1968–1971, when both cod and herring stocks were larger than during 1974–2098 (also see Lundström et al. 2007). In the same way, in the Bothnian Sea, where grey seals have also increased in number and are the main predator for herring, this marine mammal consumes 6–12% of the total herring removal annually (Gårdmark et al. 2012).

Environmental forcing and cod fishing mortality (*F*_cod_) impacted the fish biomass substantially. Similarly, MacKenzie et al. (2011) showed that grey seal predation had a lower impact on cod recovery than other factors such as salinity and fishing. Concurrently, we showed that the effect of seal abundance on fish biomass, catches and predation mortality (as Q of adult fish) is greatly modulated by the environment and the cod fishing pressure, which indicates that the Baltic Sea is very environmentally driven and, at the same time, highly sensitive to anthropogenic impacts.

The combination of *F*_cod_ = 1 and the Env2 scenarios yielded the lowest cod biomass of all the analysed future scenarios, regardless of the seal abundance. Similarly, Niiranen et al. (2013) described that under a Env2-like scenario combined with *F*_cod_ = 1.1, cod presented the lowest biomass, whereas clupeid biomasses were higher. This means that there might be a synergistic negative interaction between high nutrient and high-temperature values (Env2) in combination with a high *F*_cod_ affecting cod biomass growth. However, we found that the overall fish biomass under the Env0 scenario, particularly when *F*_cod_ = 0.3, was the lowest. This is probably due to decreased primary production as a result of decreased nutrient inputs to the Baltic Sea assumed in Env0, which offsets the positive effects of decreased hypoxia (Fig. S2). However, we note that our model may overestimate the positive effects of a high primary production in Env1 and Env2, as under eutrophic conditions, especially when combined with high temperatures, the primary producer community may shift towards unfavorable species for consumers (Lehtiniemi et al. 2002; Neumann et al. 2012; Suikkanen et al. 2013).

The significantly higher MTI of seals on cod compared to the impact by cod fisheries indicated that the major driver of cod biomass in the Baltic Sea is the fishery. Similarly, Eero et al. (2015) documented that among fishing mortality, nutrient concentration, climate-driven hydrographic conditions and seal abundance, the latter was the only factor that did not have detrimental effects on the Eastern Baltic cod spawning stock biomass. Moreover, the negative MTI of cod fishery on the adult seal group due to removal of the seals’ prey could lead to a deleterious effect on the Baltic seal populations, especially given the current situation of decreasing seal health condition (HELCOM 2017), which can be interpreted as an early signal of density dependence (Harding et al. 2018). Even though food limitation of seals is not plausible at current fish and seal population sizes, the lack of cod may affect seal populations in the future. It is also worth mentioning that the positive MTI values of the adult seal group on adult sprat and herring during the simulated period is likely linked to the impact that seals have on cod, which are in turn the main predators for herring and sprat, suggesting a trophic cascade effect. Our results also showed that predation mortality of Baltic fish by grey seals was higher in the Env0 scenario, in which cod, sprat and herring abundances had their lowest values. Thus, maintaining cod populations at relatively high abundance levels could reduce cod predation mortality by seals (e.g., Hammill et al. (2014)). Interestingly, the differences in seal diet composition between the datasets used in our model showed a decrease in the consumption of larger prey like cod and an increase in smaller prey (e.g. herring). This suggests a decrease in the trophic level of the seal diet, which coincides with the findings by Hanson et al. (2017) using stable isotopes of grey seals in the North Sea.

Our simulations have certain limitations as the model is a simplification of the food web in the open waters of the Baltic Proper (for further details see also Appendix S4). For example, changes in *F*_herring_ and *F*_sprat_ could have been defined, but at the expenses of further complexity to the study and a significantly larger amount of simulations. This subject is worthy of further study, as disproportionately decreasing F of herring or sprat compared to the other species could increase their populations and, consequently, their role in the diet of seals. Also, the model lacks some of the components of the system that are economically important in some areas of the Baltic Sea, such as sea trout, whitefish, flatfish, eels and perch, among others. We found that a more negative MTI of seals on their modelled prey could be expected if seal consumed exclusively herring, sprat and cod within the studied system (i.e. redistributing all the ‘import’ diet proportionally into the modelled prey) (Fig. S7), suggesting that it might be worth including coastal fish stocks in future models. Hansson et al. (2017) showed that seals might have a significant impact on some coastal fish populations, although the authors acknowledged that the proportions of near-shore fish species in the seals’ diet might have been overestimated, as these diet samples are generally collected in coastal areas. If we consider the same bias in our study, our projections under the extreme seal50 scenario suggest that a higher proportion of more offshore species such as cod, herring and sprat in the seals’ diet is still unlikely to have a significant impact on the offshore fish stocks. A spatially explicit model of seal–fishery interactions would be needed to more thoroughly investigate the impacts of seals on both coastal and offshore fish populations. A spatially explicit model would allow an explicit modelling of seal biomass development dependent on their interactions with both coastal and offshore fish species and fisheries. In addition, including the coastal perspective in the model could facilitate the assessment of the operational impacts that seals have on small-scale fisheries (Varjopuro 2011).

Most of the operational interactions between seals and fisheries take place in coastal areas. Given the ecological and oceanographic differences between the open sea and the coastal areas, our model does not allow to extrapolate our results in order to elucidate the ecological effects of seals on Baltic coastal fisheries such as salmon, eel or whitefish. Nevertheless, the operational conflicts between seals and coastal fisheries should be managed following an approach that can both secure the revenues of the fishers and guarantee the conservation and good status of the grey seal population in the Baltic Sea. As an example, in Sweden some studies have shown the efficacy of new seal-proof fishing traps in the Baltic (Königson 2011).

## Conclusions

This work shows that the impacts of the increasing Baltic grey seal population on fish stocks are complex. We emphasize the need to consider a range of possible ecosystem contexts when evaluating potential impacts of top predators. Our results provide evidence that consumption by grey seals at a population size of ~ 30 000 individuals affects fish biomass in the offshore Baltic Proper significantly less than climate change, nutrient load and fisheries. Responses of fisheries stakeholders to a further increase in the seal population are not easy to foresee but negative responses among some fisheries collectives can be anticipated. However, we suggest that an increasing seal population is not likely to hinder the preservation of the main Baltic fish stocks, and we expect that the outcomes of our study will help to shed light on the controversy.

Conflicts and competition for fisheries resources between humans and marine predators are difficult to quantify, and are therefore challenging to manage. Our study can serve as a guide for more holistic approaches to management and conservation advice. When managing fisheries, it is necessary to consider not only the state of the fish stocks but also the environmental conditions and the biology of the fish predators, as well as the fisheries response to these factors, in an integrative way. Moreover, the management and conservation of seals need to be strategic and based on long-term plans.

## Electronic supplementary material

Below is the link to the electronic supplementary material.

Supplementary material 1 (PDF 2466 kb)
