# Supplementary material for: The necessity of a holistic approach when managing marine mammal–fisheries interactions: Environment and fisheries impact are stronger than seal predation
Source: Ambio. 2018 Dec 8;48(6):552–64. doi: 10.1007/s13280-018-1131-y (PMC6486897; doi:10.1007/s13280-018-1131-y)
Supplement: Supplementary file 1 — Supplementary material 1 (PDF 2466 kb) [file 13280_2018_1131_MOESM1_ESM.pdf]

Ambio

Electronic Supplementary Material

Title: **The necessity of a holistic approach when managing marine mammal–fisheries interactions: Environment and fisheries impact are stronger than seal predation**

Authors: David Costalago, Barbara Bauer, Maciej T. Tomczak, Karl Lundström, Monika Winder

Supplementary information.

Appendix S1. Baltic Proper Ecopath model update.

Departing from the existing Baltic Proper Ecopath model built to describe the Baltic open sea area food-web dynamics between 1974-2006 (Tomczak et al. 2012; Tomczak et al. 2013), we divided the seal group into juvenile and adult groups using the 'multi-stanza' representation (Gales et al. 2003; Walters et al. 2010) and defined new Ecopath parameters and diet composition for these two groups. The split was necessary as juveniles and adults of grey seal have different energetic requirements (Innes et al. 1987; Hammill and Stenson 2000) and their diets differ in the prey size and proportion (Lundström et al. 2007; Lundström et al. 2010; Hanson et al. 2017). We derived population biomass estimations from the number of counted seals during four decades (1975-2015) of surveys obtained from the Baltic Marine Environment Protection Commission (HELCOM) and from the Swedish Museum of Natural History (SMNH). We assumed that the number of counted seals in the monitoring reported by HELCOM was 85% of the actual population size (Hiby et al. 2007) and that the distribution during monitoring (grey seal molting period, i.e. May-June) was representative for the entire year. It was assumed that one third of the counted grey seal individuals in the Baltic Sea corresponds to juveniles and the rest to adults (Hårding et al. 2013). The average weight of a grey seal juvenile as considered for the model (i.e. < 60 months old) is 100 kg (Innes et al. 1987), while the weight range of a female adult is 100-180 kg and of a male adult is 180-300 kg (Perrin et al. 2009); thus, we considered the average weight of an adult in the model to be 200 kg. We included new data on the diet and biological parameters corresponding to grey seals from the North Atlantic (data from the Baltic seals were preferentially used, when available; Table S1).

The Electivity (selection index) describes a predator's preference for prey. It scales from -1 to 1; where -1 indicates total avoidance of a prey; 0 indicates that a prey is taken in proportion to its abundance in the ecosystem; and 1 indicates total preference for a prey. The electivity index as displayed in Fig. S1 is the standardized forage ratio of Chesson (1983).

The ecotrophic efficiency (*EE*) is a dimensionless parameter representing the fraction of the production that is used in the system, i.e. either passed up the food web, used for biomass accumulation, migration or export. Ecotrophic efficiency varies between 0 and 1 and can be expected to approach 1 for groups with considerable predation pressure.

Appendix S2. Ecosim parameters.

The 'Group info' parameters for the seal groups (Table S3) were adjusted so the feeding effects of the seals were maximized. Seals do not need to limit the time spent feeding to avoid predation in the Baltic, thus we gave 'maximum relative feeding time' values higher than the default value of 2 (meaning than that feeding time may double when prey became more scarce), i.e. 5 for the juvenile seal stanza and 10 for the adult seal stanza. As usually recommended for marine mammals, we set the 'feeding time adjustment rate' to 0.5, meaning that seals will adjust their foraging time to some extent based on food availability, to reach 'normal' individual consumption rates, i.e. as under conditions when the Ecopath model was estimated. QBmax/QBo values represent the upper limit of individual intake rates and are by default set to 1000 in Ecosim

models (i.e., predators are not limited by handling times and can increase their intake greatly when prey densities increase). Here this value was set at 500, although preliminary tests did not show any differences in biomass projected with QBmax/QBo values between 100-1000, which is not surprising as this parameter only becomes relevant at a particular combination of very low predator density, very high prey density and high prey vulnerability. We set the 'predator effect on feeding time' parameter for seals to 0 (that is, no effect). Grey seals are considered opportunistic and thus they are thought to feed predominantly on the more available prey species (Murie and Lavigne 1992; Mark Jessopp et al. 2013).

In Ecosim, the temporal changes in the flow rates between available and unavailable prey behavioral states are calculated based on the "foraging arena" concept and are regulated by the predator- and prey-specific vulnerability parameters. Ecosim users can specify vulnerability multipliers (k_{ij}) which determine how much maximum predation mortality rate of prey *i* can increase with an increasing biomass of predator *j*. When vulnerability multipliers are high ($k_{ij} \gg 2$), the predators can increase their consumption of prey when their biomass increases, while at low values ($k_{ij} = 1$) total prey consumption rates stay close to their Ecopath rates even when predator biomass increases. The former situation is usually referred to as 'top-down control' of the trophic interaction in EwE models and typically occurs when the prey has no refuge from predation and/or the predator is far from carrying capacity in the year which the Ecopath model describes and thus, has a large potential to increase its predation pressure on prey (Christensen and Walters 2004). Both criteria are fulfilled in the case of grey seals and their fish prey. Thus, we specified a value of $k_{ij} = 100$ for this parameter for both juvenile and adult seals.

Appendix S3. Mixed Trophic Impact (MTI).

To estimate the direct and indirect effects (positive or negative) that changes of the biomass of one functional group might have on another, Ecopath uses the Network Analysis routine called Mixed Trophic Impact (MTI). It synthesizes all the effects that an infinitesimal change in the biomass of a group will have on the biomass of other groups in a system (Ulanowicz and Puccia 1990) and gives an ecosystem overview of the trophic interactions.

It is calculated from the difference between the proportion that group *i* contributes to the diet of group *j*, and the proportion that group *i* takes from the production of group *j* (Christensen and Walters 2004):

$$MTI_{ij} = DC_{ij} - FC_{j,i}$$

where DC_{ij} is the diet composition term expressing how much *j* contributes to the diet of *i*, and $FC_{j,i}$ is a host composition term giving the proportion of the predation on *j* that is due to *i* as a predator (or fishery).

In this study, only 26 out of the total 124 Ecopath models generated by the 'Ecopath from Ecosim' routine were not mass-balanced (years 1978, 1979, 1985, 1987, 1991, 1996, 1997, 1999, 2000, 2005, 2010, 2011, 2014, 2016, 2019, 2033, 2034, 2036, 2038, 2041, 2044, 2050, 2056, 2059, 2062, 2068, 2079, 2082, 2088, 2091, 2093, 2095 and 2098). Although these 26 models were slightly unbalanced (the Ecotrophic Efficiencies (EEs) of the unbalanced groups in most of these models were <2), we removed those years from the MTI estimations.

Appendix S4. Assumptions and limitations of the model.

The main assumption in Ecopath and Ecosim models is that the modeled functional groups are related to the main components of the food-web. However, this structure is only a simplification of the ecosystem and not all food-web components are included in the model and thus these models present certain limitations. For instance, Plagányi and Butterworth (2004) discussed the implications of the foraging arena hypothesis. Furthermore, Aydin (2004) explored the implications of the “fixed growth efficiency” function in Ecosim represented by the P/Q rates, which implies that the model does not adequately account for changes in population energetics as a population’s size and structure changes due to heavy fishing or release of fishing pressure. Also, as Coll et al. (2009) mentioned, Ecosim is dependent on the mass-balance (equilibrium) assumption of Ecopath. This infers the risk of encountering errors in the interpretation of the Ecosim results, when these are extrapolated far beyond the equilibrium for which Ecopath data are available (Mackinson et al. 1997; Walters et al. 1997). Moreover, Ecopath and Ecosim models present limitations when interpreting the outcomes for highly migratory species or stocks patchiness (see Christensen and Walters (2004)), such as herring and sprat. Thus, when interpreting the simulations, we carefully considered the assumptions and limitations underlying Ecopath and Ecosim models. Assumptions and limitations of the model used for this study in particular are discussed in detail in Tomczak et al. (2012). Moreover, Niiranen et al. (2012) conducted a study to specifically assess the uncertainties and sensitivity of the BaltProWeb model, in which our model is based. In their study, Niiranen et al. (2012) also assessed the potential uncertainty under different future conditions with the same fishery and climate scenarios as in our model.

Table S1. Seal parameters and input data sources. B = Biomass (t km^{-2}), P/B = Production/Biomass or Total Mortality (Per year), Q = Consumption (t km^{-2} per year), *k* = von Bertalanffy curvature parameter (Per year), BA = Biomass accumulation rate (t km^{-2} per year), W_{mat} = weight at maturity (kg), W_{inf} = weight at infinity (kg).

	Parameter	Value	Source
	k	0.170	sealifebase.ca
	BA/B	0.010	Unpublished data from Olle Karlsson, Swedish Museum of Natural History
	W_{mat}/W_{inf}	0.090	Hårding et al. (2015), sealifebase.ca
Adults (age >5 y.o.)	B	0.00146	Unpublished data from Olle Karlsson, Swedish Museum of Natural History
	P/B	0.110	Harvey et al. (2003)
	Q/B	4.959	Lundström (2012)
Juveniles (age 0-5 y.o.)	B	0.000112	Unpublished data from Olle Karlsson, Swedish Museum of Natural History
	P/B	0.202	Harvey et al. (2003)
	Q/B	10.160	Lundström (2012)

Table S2. Basic estimates obtained after the Ecopath model was mass-balanced. These are output indices for assessing the status of the overall ecosystem and of its constituent groups.

Group name	Trophic level	Biomass (t km^{-2})	Production / biomass (/year)	Consumption / biomass (/year)	Ecotrophic Efficiency
Cyanobacteria	1.0000	3.1213	150.3635	0.0000	0.0604
Phytoplankton	1.0000	8.1197	142.6500	0.0000	0.6043
Microzooplankton	2.0000	2.7928	97.1298	336.1954	0.4402
Temora sp.	2.2000	1.7626	20.7866	54.5046	0.7947
Acartia sp.	2.2000	1.3459	22.1911	69.4572	0.5813
Pseudocalanus sp.	2.3000	3.4631	8.6097	26.3624	0.7585
Other mesozooplankton	2.2000	3.6719	19.2671	73.4631	0.1830
Mysids	2.5000	2.4600	5.8296	21.9605	0.5136
Meiozoobenthos	2.0000	4.6872	5.5354	31.8693	0.7407
Macrozoobenthos	2.0979	27.3841	1.9800	10.7310	0.2340
Juvenile Sprat	3.2201	1.4079	1.9400	11.4240	0.3056
Adult Sprat	3.2310	4.1418	0.8624	5.2301	0.4626
Juvenile Herring	3.2841	5.1766	1.9907	5.0750	0.1830
Adult Herring	3.2980	5.5435	0.4772	2.1128	0.5633
Cod larvae	3.2800	0.0002	0.5341	73.9923	0.0000
Juvenile Cod	3.3426	0.1074	1.2759	12.7022	0.0554
Small Cod	3.8212	0.6049	0.5814	5.8072	0.6144
Adult Cod	4.0119	0.5185	1.1146	3.8950	0.6468
Juvenile Seal	4.4926	0.0000	0.2021	35.9665	0.0000
Adult Seal	4.5636	0.0015	0.1097	4.9590	0.0000
Detritus (water column)	1.0000	3255.8100			0.8166
Detritus (sediment)	1.0000	4651.1700			0.5433

Table S3. Group info values. The Group info form enables entry of these Ecosim parameters for all scenarios.

Group name	Max. relative P/B	Max. relative feeding time	Feeding time adjust rate [0,1]	Fraction of other mortality sensitivity to changes in feeding time	Predator effect on feeding time [0,1]	Density-dep. catchability: Qmax/Qo [>=1]	QBmax/QBo (for handling time) [>1]	Switching power parameter [0,2]
Cyanobacteria	2							
Phytoplankton	2							
Microzooplankton		2	0	1	0	1	1000	0
Temora sp.		2	0	1	0	1	1000	0
Acartia sp.		2	0	1	0	1	1000	0
Pseudocalanus sp.		2	0	1	0	1	1000	0
Other mesozooplankton		2	0	1	0	1	1000	0
Mysids		2	0	1	0	1	1000	0
Meiozoobenthos		2	0	1	0	1	1000	0
Macrozoobenthos		2	0	1	0	1	1000	0
Juvenile Sprat		2	0.5	1	0	3	1000	0
Adult Sprat		2	0	1	1	4	1000	0
Juvenile Herring		2	0.5	1	0	2	1000	0
Adult Herring		2	0	1	0	3	1000	0
Cod larvae		2	0.5	1	0	2	1000	0
Juvenile Cod		2	0.5	1	0	1	1000	0
Small Cod		2	0.5	1	0	1	1000	0
Adult Cod		2	0	1	0	1	1000	0
Juvenile Seal		5	0.5	1	0	1	500	2
Adult Seal		10	0.5	1	0	1	500	2

Table S4. Diet matrix composition for the Ecopath baseline model (1974). Predator groups in columns and prey groups in rows.

Prey group	Microzooplankton	*Temora* sp.	*Acartia* sp.	*Pseudocalanus* sp.	Other mesozooplankton	Mysids	Meiozoobenthos	Macrozoobenthos
Cyanobacteria	0.001	0.050	0.050	0.050	0.050			
Phytoplankton	0.450	0.550	0.400	0.400	0.500	0.300		
Microzooplankton		0.200	0.200	0.300	0.200			
Temora sp.						0.150		
Acartia sp.						0.100		
Pseudocalanus sp.						0.050		
Other mesozooplankton						0.050		
Mysids								
Meiozoobenthos								0.065
Macrozoobenthos								0.030
Juvenile Sprat								
Adult Sprat								
Juvenile Herring								
Adult Herring								
Cod larvae								
Juvenile Cod								
Small Cod								
Adult Cod								
Juvenile Seal								
Adult Seal								
Detritus (water column)	0.549	0.200	0.350	0.250	0.250			
Detritus (sediment)						0.200	1.000	0.905
Import						0.150		

Table S4. Diet matrix composition for the Ecopath baseline model (1974). Predator groups in columns and prey groups in rows. (Continued)

Prey group	Macrozoobenthos	Juv. Sprat	Juv. Sprat	Ad. Sprat	Juv. Herring	Ad. Herring	Cod larvae	Juv. Cod	Small Cod	Ad. Cod	Juv. Grey Seal	Ad. Grey Seal
Cyanobacteria												
Phytoplankton												
Microzooplankton												
Temora sp.		0.330	0.330	0.353	0.240	0.159	0.100					
Acartia sp.		0.300	0.300	0.138	0.140	0.043	0.100					
Pseudocalanus sp.		0.199	0.199	0.307	0.230	0.356	0.800					
Other mesozooplankton		0.171	0.171	0.200	0.100	0.047						
Mysids		0.001	0.001	0.001	0.170	0.150		0.250	0.150	0.033		
Meiozoobenthos	0.065											
Macrozoobenthos	0.030					0.047		0.536	0.265	0.168		
Juvenile Sprat						0.003		0.008	0.151	0.093	0.024	
Adult Sprat								0.037	0.136	0.151	0.062	
Juvenile Herring								0.049	0.213	0.267	0.189	0.173
Adult Herring								0.006	0.051	0.143	0.187	0.171
Cod larvae												
Juvenile Cod									0.002	0.001	0.035	0.041
Small Cod										0.005	0.179	0.213
Adult Cod											0.087	0.107
Juvenile Seal												
Adult Seal												
Detritus (water column)												
Detritus (sediment)	0.905											
Import				0.001	0.120	0.195		0.114	0.032	0.139	0.236	0.295

Table S5. Biomass (t km^{-2}).

		Adult cod				Adult herring				Adult sprat			
		F_{cod} = 0.3		F_{cod} = 1		F_{cod} = 0.3		F_{cod} = 1		F_{cod} = 0.3		F_{cod} = 1	
Environmental scenario	Seal scenario	Mean	SD	Mean	SD	Mean	SD	Mean	SD	Mean	SD	Mean	SD
Env0	seal0	1.21	0.18	0.49	0.14	4.95	0.84	5.73	0.68	0.77	0.79	2.33	0.6
	seal1	1.14	0.18	0.49	0.14	5	0.82	5.7	0.65	0.86	0.8	2.43	0.56
	seal50	0.92	0.14	0.48	0.14	5.05	0.73	5.59	0.63	1.26	0.67	2.73	0.44
Env1	seal0	3.07	0.74	0.7	0.2	10.57	2.16	13.34	2.71	2.81	0.68	4.81	0.69
	seal1	3	0.74	0.68	0.2	10.61	2.16	13.34	2.71	2.87	0.67	4.85	0.68
	seal50	2.76	0.68	0.63	0.18	10.72	2.16	13.3	2.75	3.06	0.66	4.95	0.7
Env2	seal0	2.7	0.89	0.34	0.15	12.79	4.48	16.35	5.55	6.13	1.76	7.77	1.83
	seal1	2.64	0.89	0.34	0.15	13.24	4.56	16.3	5.55	6.12	1.72	7.79	1.82
	seal50	2.36	0.81	0.33	0.16	13.09	4.58	16.49	5.86	6.31	1.73	7.73	1.74

Table S6. Fish consumption by seals (Q) in t km^{-2}.

		Adult cod				Adult herring				Adult sprat			
		F_{cod} = 0.3		F_{cod} = 1		F_{cod} = 0.3		F_{cod} = 1		F_{cod} = 0.3		F_{cod} = 1	
Environmental scenario	Seal scenario	Mean	SD	Mean	SD	Mean	SD	Mean	SD	Mean	SD	Mean	SD
Env0	seal1	0.18	0.04	0.08	0	0.03	0.02	0.16	0.05	0	0	0.01	0
	seal50	0.51	0.13	0.02	0.02	0.25	0.13	0.71	0.21	0	0	0.09	0.03
Env1	seal1	0.35	0.06	0.02	0	0.03	0.01	0.19	0.04	0	0	0	0
	seal50	1.25	0.4	0.07	0.03	0.19	0.08	0.87	0.29	0	0	0.04	0.02
Env2	seal1	0.3	0.09	0	0	0.07	0.03	0.23	0.05	0	0	0.02	0.01
	seal50	1.02	0.43	0.01	0.01	0.41	0.21	0.99	0.34	0.04	0.02	0.1	0.05

Table S7. Catches (t km^{-2}).

		Adult cod				Adult herring				Adult sprat			
		F_{cod} = 0.3		F_{cod} = 1		F_{cod} = 0.3		F_{cod} = 1		F_{cod} = 0.3		F_{cod} = 1	
Environmental scenario	Seal scenario	Mean	SD	Mean	SD	Mean	SD	Mean	SD	Mean	SD	Mean	SD
Env0	seal0	0.36	0.06	0.26	0.09	0.54	0.09	0.62	0.07	0.16	0.16	0.49	0.13
	seal1	0.34	0.05	0.23	0.08	0.55	0.09	0.62	0.07	0.18	0.17	0.51	0.12
	seal50	0.28	0.04	0.17	0.09	0.55	0.08	0.61	0.07	0.26	0.14	0.57	0.09
Env1	seal0	0.92	0.22	0.7	0.2	1.15	0.24	1.45	0.3	0.59	0.14	1	0.14
	seal1	0.9	0.22	0.68	0.2	1.16	0.23	1.45	0.3	0.6	0.14	1.01	0.14
	seal50	0.83	0.2	0.63	0.18	1.17	0.24	1.45	0.3	0.64	0.14	1.03	0.15
Env2	seal0	0.81	0.27	0.34	0.15	1.39	0.49	1.78	0.61	1.28	0.37	1.62	0.38
	seal1	0.79	0.27	0.34	0.15	1.44	0.5	1.78	0.61	1.28	0.36	1.63	0.38
	seal50	0.71	0.24	0.33	0.16	1.43	0.5	1.8	0.64	1.32	0.36	1.62	0.36

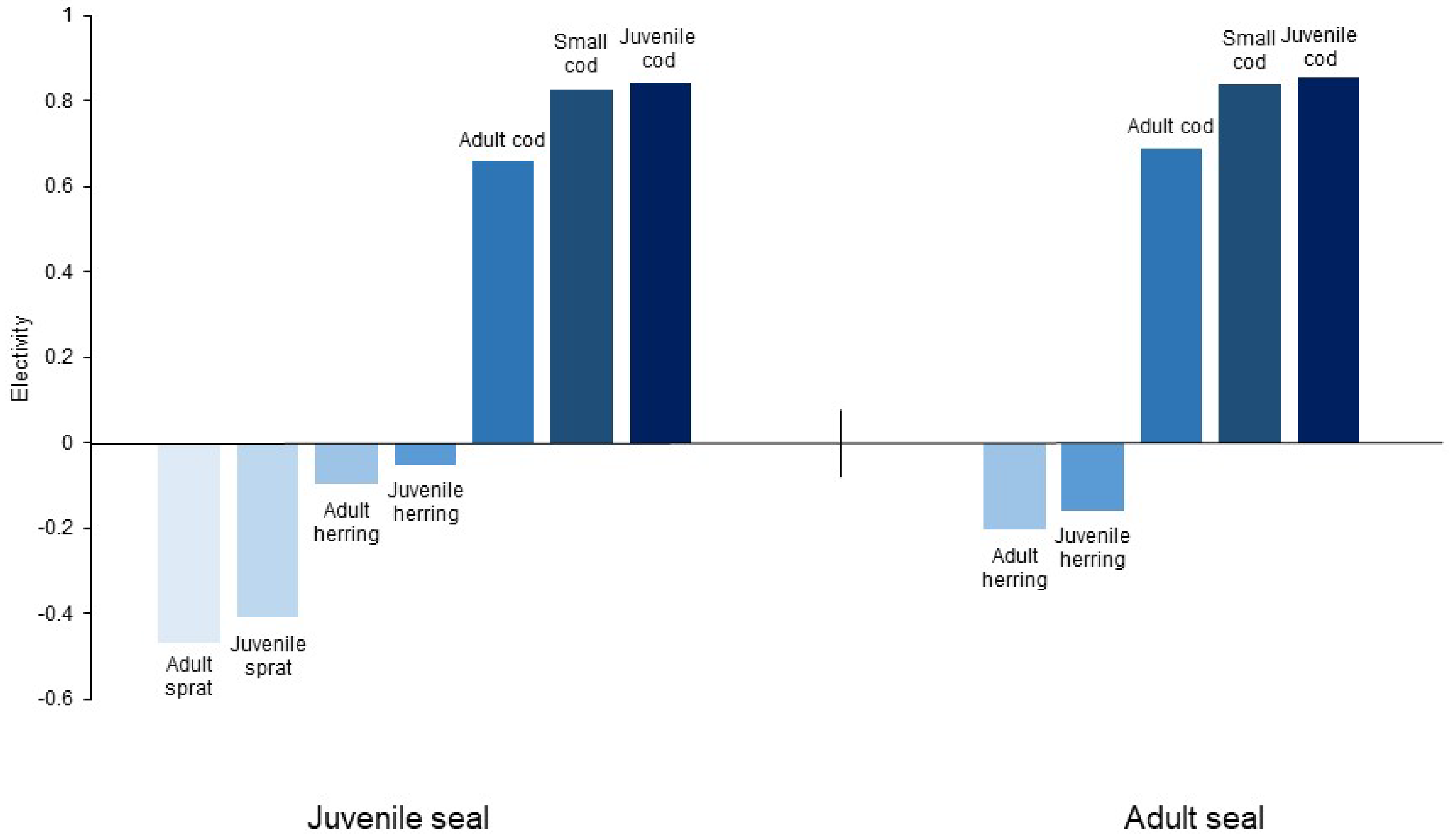

Figure S1. Electivity index showing seal's preference for the modelled prey. It scales from -1 to 1; where -1 indicates total avoidance of a prey; 0 indicates that a prey is taken in proportion to its abundance in the ecosystem; and 1 indicates total preference for a prey. The electivity values are highlighted using a blue color scale. The electivity index displayed is the standardized forage ration of Chesson (1983).

A

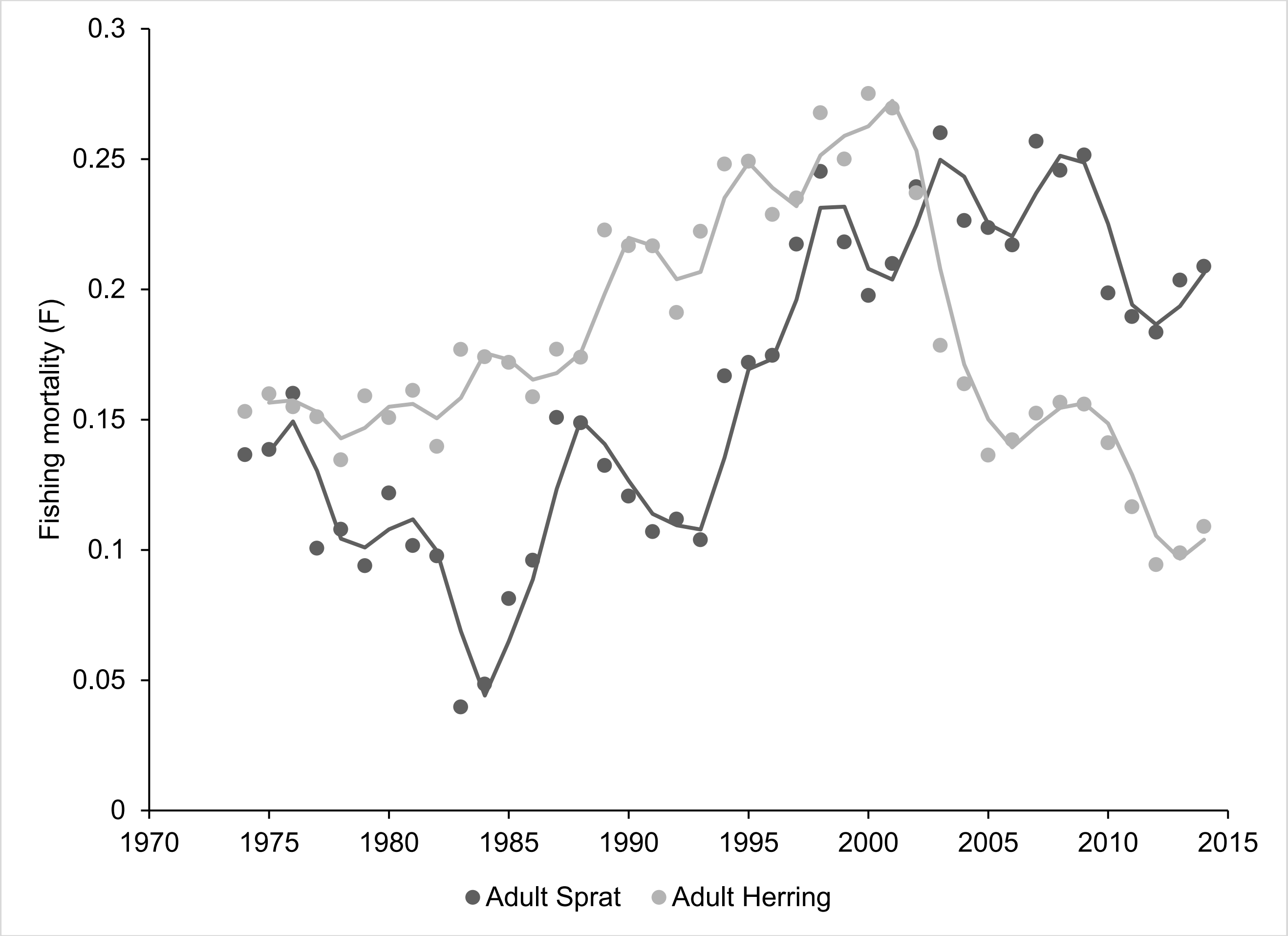

0.3
0.25
0.2
0.15
0.1
0.05
0
Fishing mortality (F)
1970
1975
1980
1985
1990
1995
2000
2005
2010
2015
Adult Sprat
Adult Herring

B

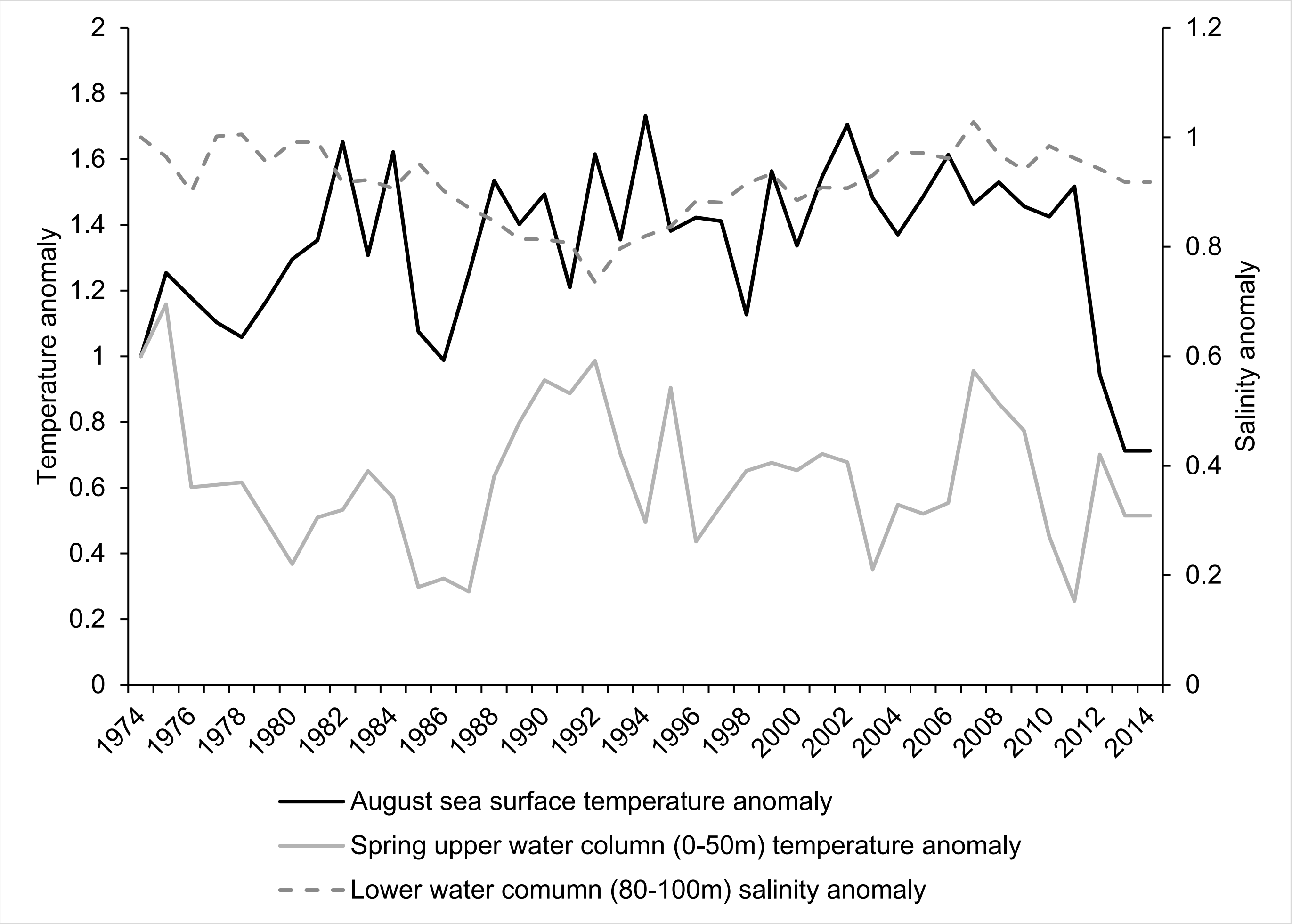
Temperature anomaly
Salinity anomaly
2
1.8
1.6
1.4
1.2
1
0.8
0.6
0.4
0.2
0
1.2
1
0.8
0.6
0.4
0.2
0
1974
1976
1978
1980
1982
1984
1986
1988
1990
1992
1994
1996
1998
2000
2002
2004
2006
2008
2010
2012
2014
August sea surface temperature anomaly
Spring upper water column (0-50m) temperature anomaly
Lower water comumn (80-100m) salinity anomaly

c

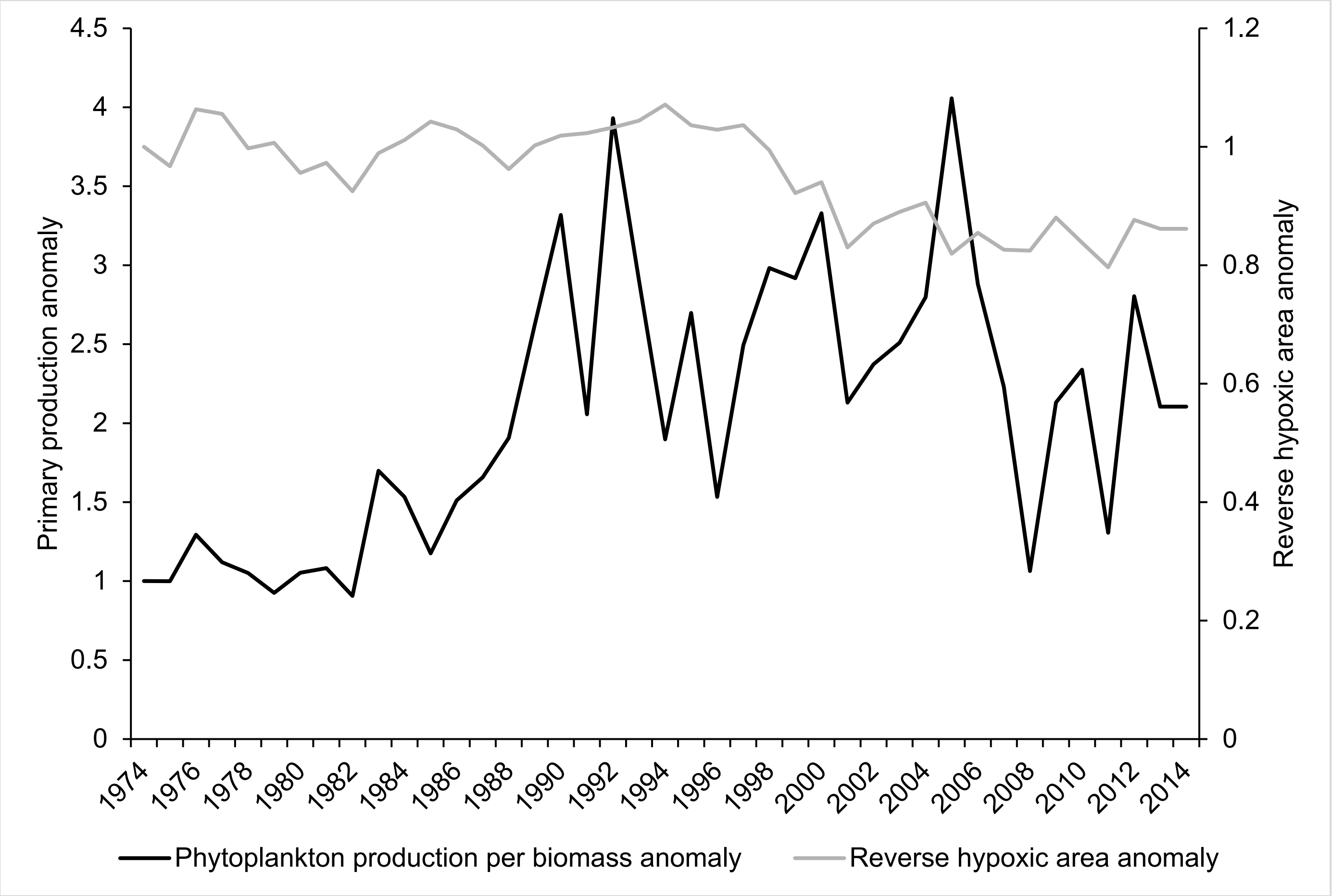
4.5
4
3.5
3
2.5
2
1.5
1
0.5
0
Primary production anomaly
1.2
1
0.8
0.6
0.4
0.2
0
Reverse hypoxic area anomaly
1974
1976
1978
1980
1982
1984
1986
1988
1990
1992
1994
1996
1998
2000
2002
2004
2006
2008
2010
2012
2014
Phytoplankton production per biomass anomaly
Reverse hypoxic area anomaly

D

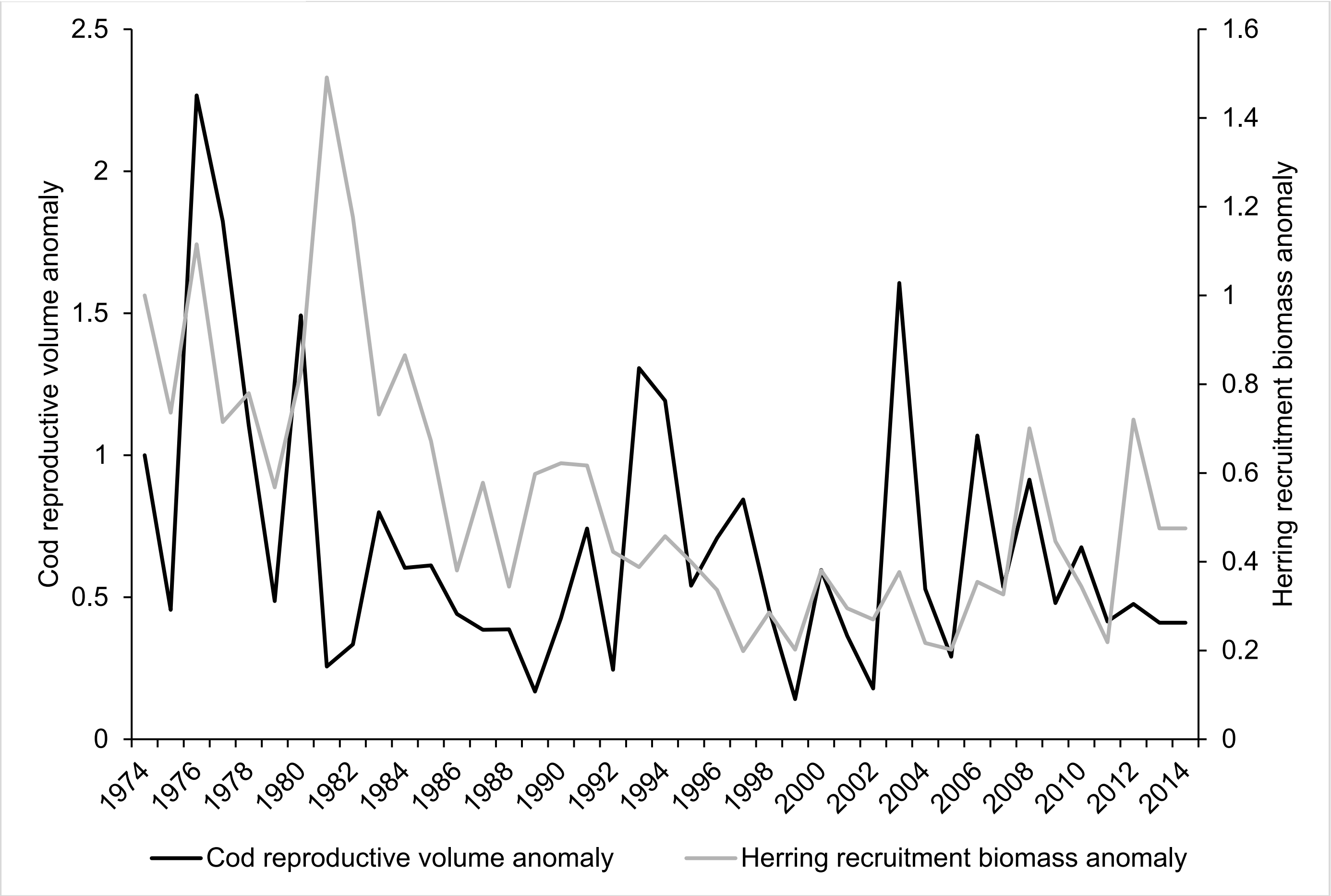

Figure S2. Forcing functions on fishing mortality (F) for adult herring and sprat (A), August and spring water temperature anomalies and salinity anomaly (B), primary production and reversed hypoxic area anomalies (C), and cod reproductive volume and herring recruitment biomass anomalies (D). See Table 1 for reference and description of the forcing functions.

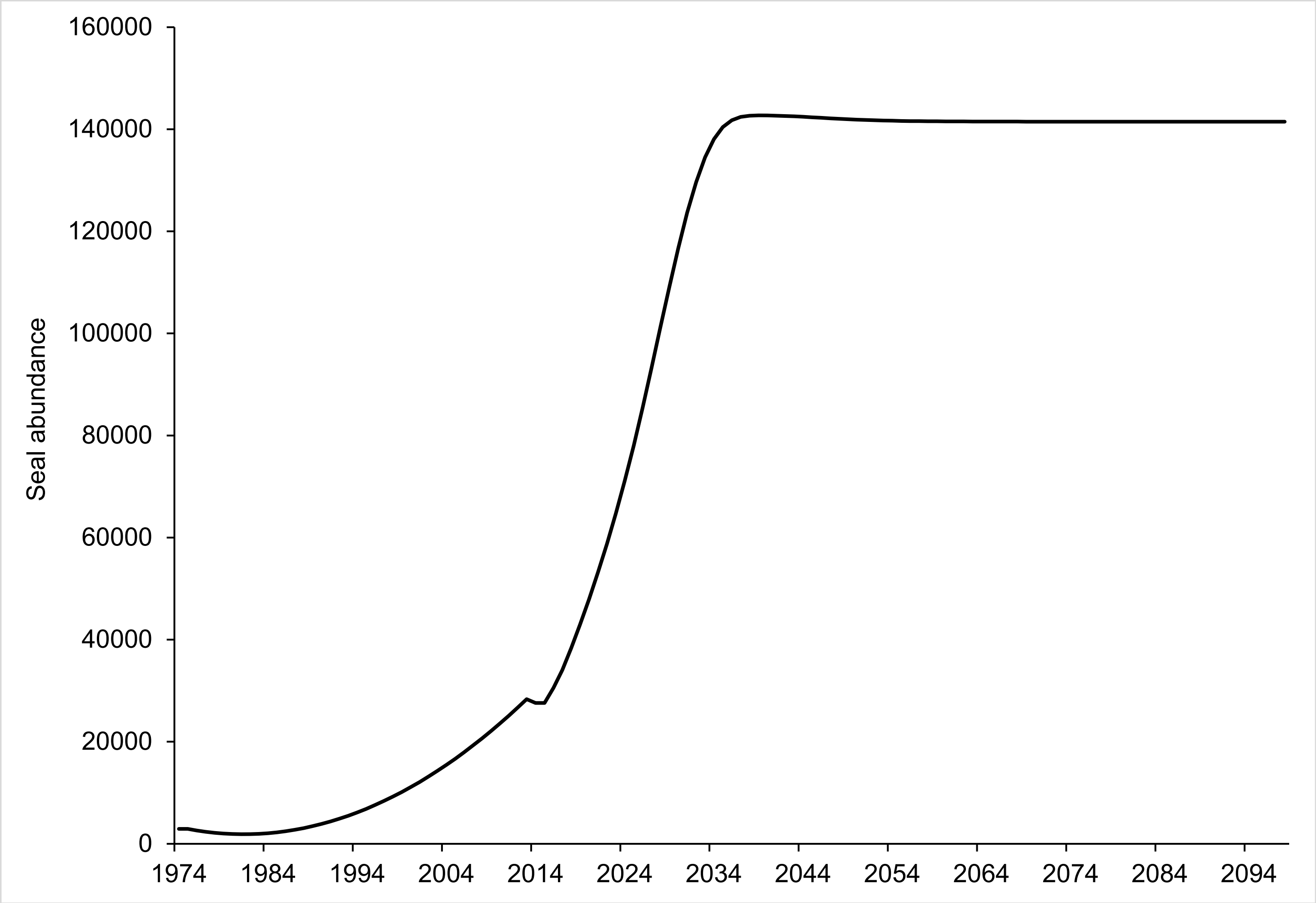

Figure S3. Seal abundance time series under the seal50 scenario. Seal biomass after 2015 was forced to grow exponentially, following the previous growth trend, until a maximum seal population size of 50 times the initial biomass from 1974 (i.e. ±140000 individuals).

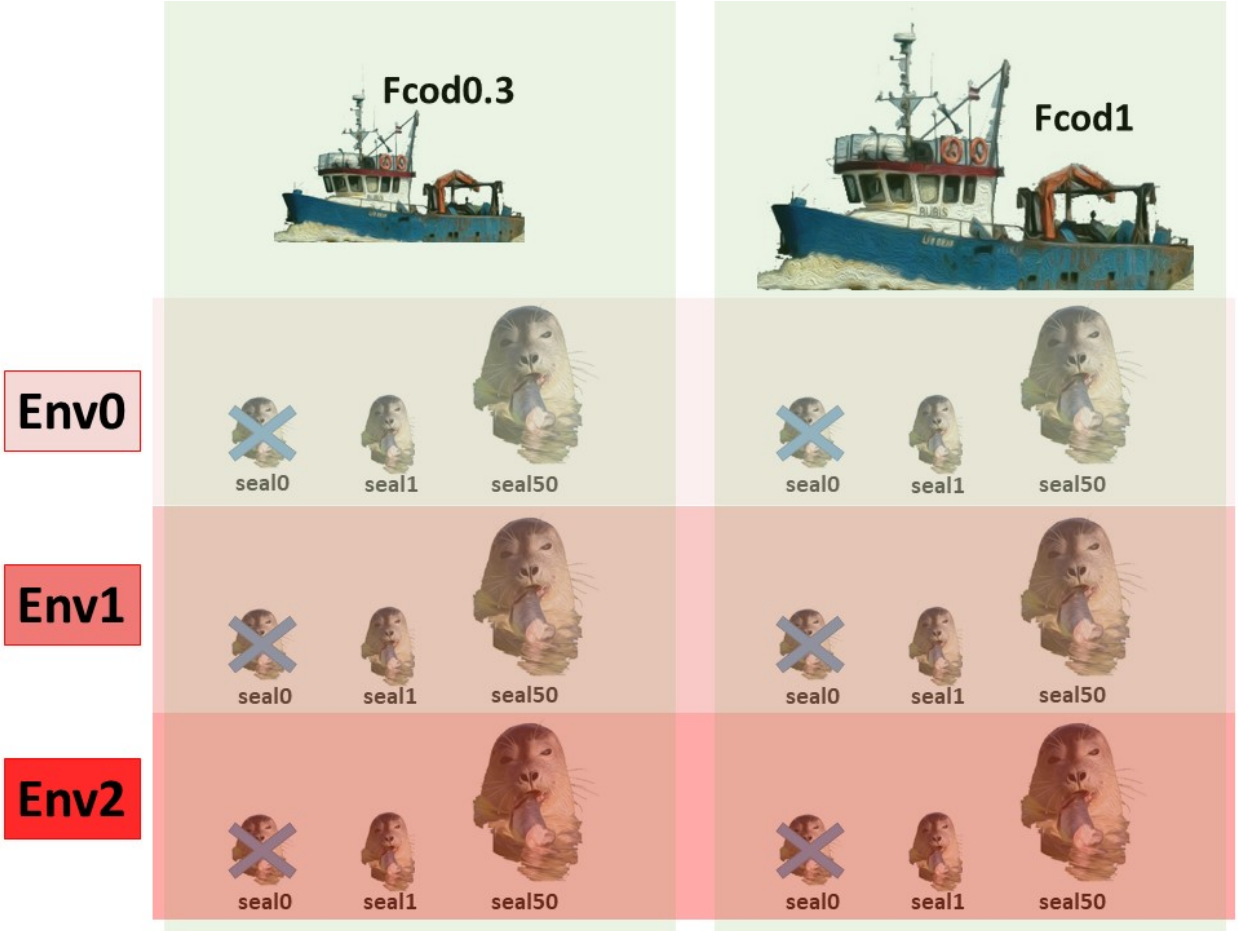

Figure S4. Combinations of seal abundance (seal0, seal, seal50), cod fishing mortality (Fcod0.3, Fcod1) and environmental (Env0, Env1, Env2) scenarios for future (2016-2098) projections. See Table 2 for reference and description of each scenario.

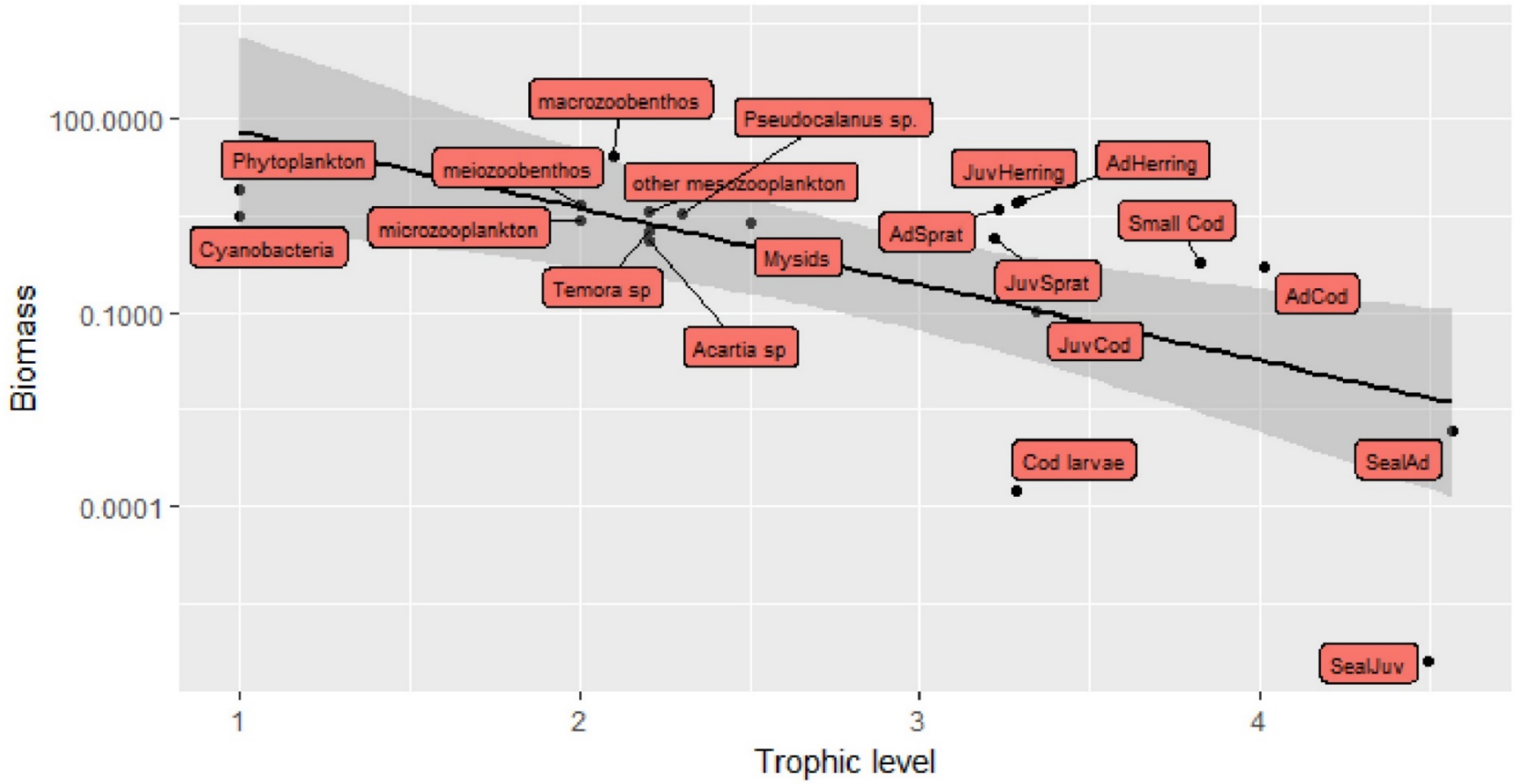

Figure S5. Pre-balance diagnostics (PREBAL) of the Baltic Proper Ecopath model plotting biomass estimates (t/km^{-2}) on a log scale with functional groups ordered by trophic level from highest to lowest. Biomass spanned five orders of magnitude. PREBAL described by Link (2010) are a way to judge the quality of Ecopath models. Pre-bal criteria state that biomass estimates and P/B and Q/B ratios should increase with decreasing trophic level. According to Link (2010), consumption by a taxon should be more than production by that taxon, as it occurs in our case. Therefore, P should not exceed Q (P/Q < 1).

A

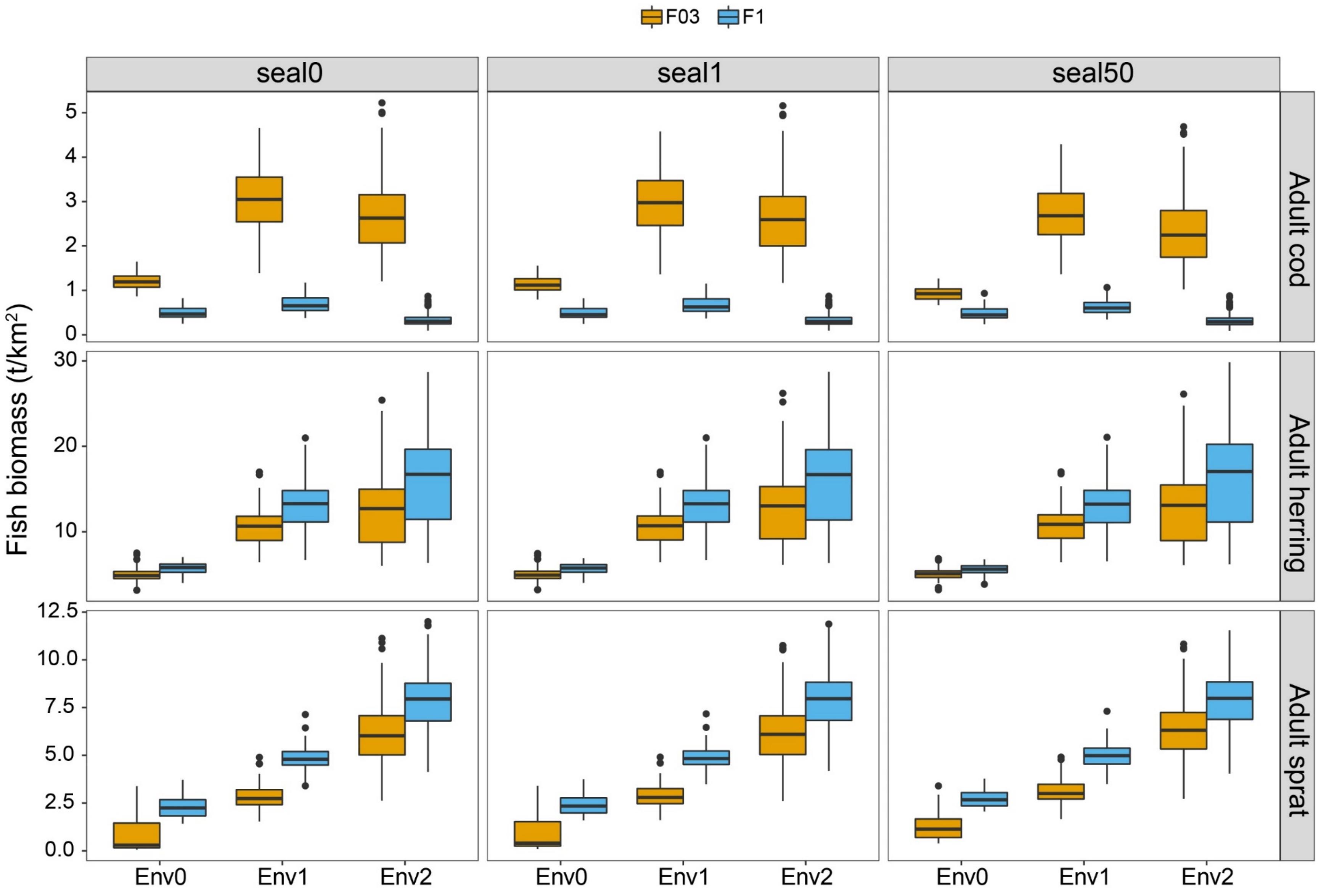

F03
F1
seal0
seal1
seal50
Adult cod
Adult herring
Adult sprat
Fish biomass (t/km²)
5
4
3
2
1
0
30
20
10
12.5
10.0
7.5
5.0
2.5
0.0
Env0
Env1
Env2

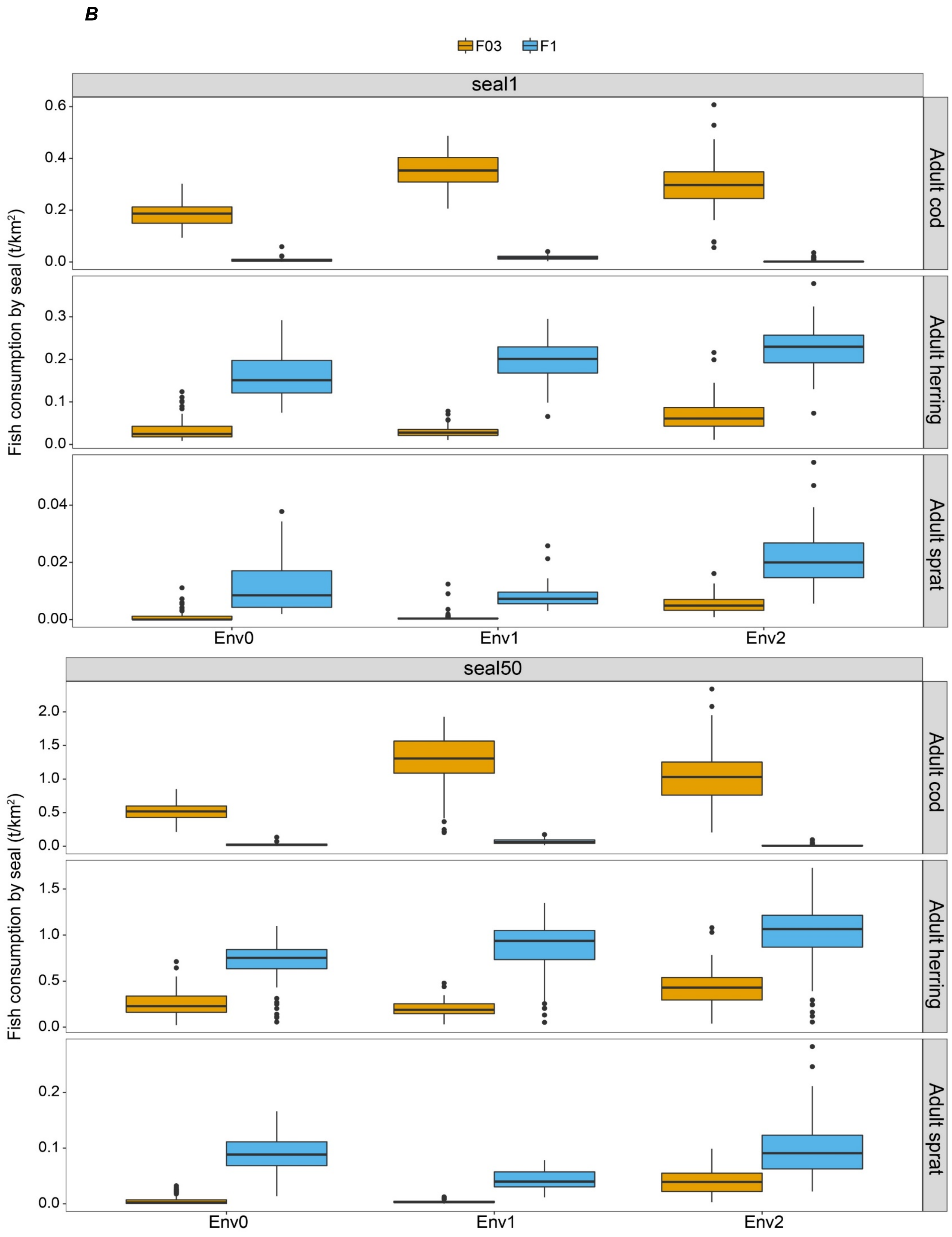
B
F03
F1
seal1
seal50
Fish consumption by seal (t/km^2)
Adult cod
Adult herring
Adult sprat
Env0
Env1
Env2
0.0
0.2
0.4
0.6
0.1
0.3
0.00
0.02
0.04
0.5
1.0
1.5
2.0

C

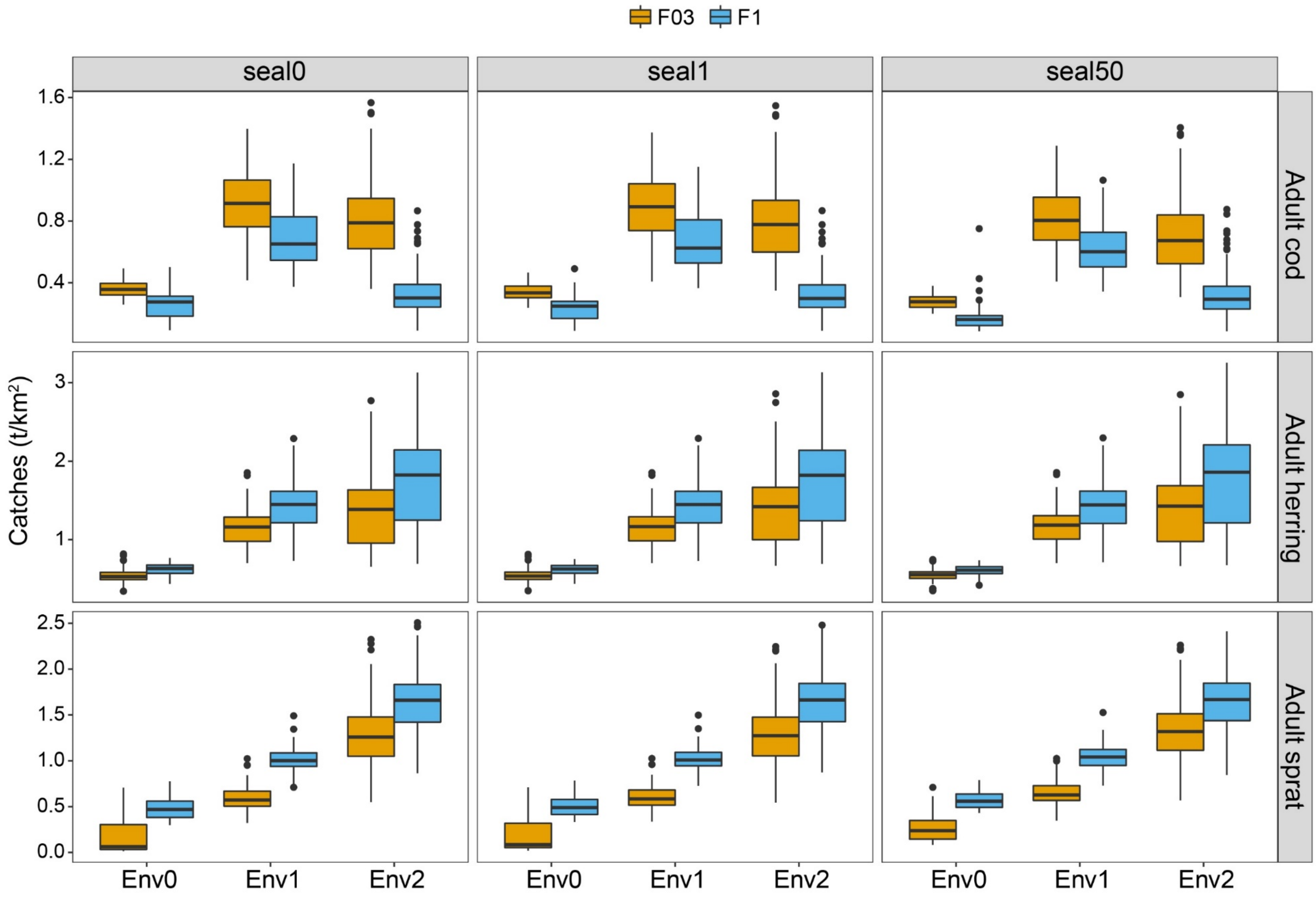

Figure S6. Biomass (A), consumption by seals (B) and catches (C) of adult cod, herring and sprat under environment scenario combinations of Env0, Env1 and Env2, seal0, seal1 and seal100 seal biomass scenarios and cod fishing mortality scenarios F_{cod} = 0.3 (orange) and F_{cod} = 1 (blue).

	JuvSprat	AdSprat	JuvHerring	AdHerring	Cod larvae	JuvCod	Small Cod	AdCod	SealJuv	SealAd	COD-Trawl	HER-Trawl	SPR-Trawl
JuvSprat	-0.156	-0.118	-0.096	-0.034	-0.065	-0.024	0.055	-0.003	-0.008	-0.007	0.011	-0.053	-0.012
AdSprat	-0.158	-0.328	-0.079	-0.048	-0.084	0.005	0.024	0.024	0.004	-0.001	0.024	-0.058	0.580
JuvHerring	-0.103	-0.080	-0.185	-0.200	-0.093	-0.110	0.052	0.101	0.119	0.104	0.090	0.107	-0.082
AdHerring	-0.030	-0.019	-0.094	-0.261	-0.094	-0.059	-0.045	0.053	0.088	0.071	0.030	0.487	-0.021
Cod larvae	0.000	0.000	0.000	0.000	0.000	0.000	0.000	0.000	0.000	0.000	0.000	0.000	0.000
JuvCod	0.008	-0.008	-0.017	0.001	0.007	-0.022	-0.011	-0.007	0.016	0.016	-0.008	-0.004	-0.007
Small Cod	-0.306	-0.060	-0.165	0.013	0.085	-0.364	-0.129	-0.152	0.039	0.043	0.084	-0.041	-0.088
AdCod	0.039	-0.048	-0.098	-0.131	0.042	-0.027	-0.394	-0.390	0.119	0.139	0.378	-0.121	-0.038
SealJuv	0.000	0.000	0.000	0.000	0.000	0.000	0.000	0.000	0.000	0.000	0.000	0.000	0.000
SealAd	0.050	0.023	0.048	0.014	-0.024	-0.194	-0.090	-0.082	-0.035	-0.039	-0.084	0.024	0.026
COD-Trawl	0.168	0.078	0.187	0.099	-0.089	0.260	-0.246	-0.400	-0.123	-0.142	-0.364	0.125	0.088
HER-Trawl	0.022	0.016	-0.047	-0.261	0.045	0.034	0.012	-0.030	-0.046	-0.038	-0.021	-0.196	0.016
SPR-Trawl	-0.018	-0.240	0.038	0.021	0.037	0.000	-0.014	-0.009	-0.001	0.001	-0.010	0.026	-0.215

	JuvSprat	AdSprat	JuvHerring	AdHerring	Cod larvae	JuvCod	Small Cod	AdCod	SealJuv	SealAd	COD-Trawl	HER-Trawl	SPR-Trawl
JuvSprat	-0.161	-0.116	-0.095	-0.030	-0.063	-0.011	0.067	0.006	0.000	-0.006	0.021	-0.049	-0.008
AdSprat	-0.166	-0.364	-0.076	-0.044	-0.090	0.016	0.027	0.023	0.015	-0.011	0.024	-0.053	0.546
JuvHerring	-0.059	-0.055	-0.168	-0.200	-0.102	-0.171	-0.009	0.094	0.147	0.145	0.068	0.100	-0.055
AdHerring	-0.007	-0.010	-0.088	-0.283	-0.076	-0.100	-0.067	0.039	0.124	0.113	0.012	0.483	-0.010
Cod larvae	0.000	0.000	0.000	0.000	0.000	0.000	0.000	0.000	0.000	0.000	0.000	0.000	0.000
JuvCod	0.016	-0.007	-0.018	0.001	0.007	-0.045	-0.028	-0.009	0.034	0.041	-0.014	-0.005	-0.004
Small Cod	-0.298	-0.043	-0.157	0.013	0.078	-0.256	-0.173	-0.169	0.117	0.162	0.084	-0.036	-0.071
AdCod	-0.020	-0.045	-0.121	-0.095	0.044	-0.020	-0.255	-0.369	0.003	0.019	0.406	-0.103	-0.042
SealJuv	0.014	0.003	0.009	-0.002	-0.004	-0.052	-0.033	-0.007	-0.010	-0.013	-0.014	0.001	0.004
SealAd	0.131	0.035	0.084	-0.015	-0.042	-0.454	-0.297	-0.057	-0.085	-0.113	-0.118	0.014	0.046
COD-Trawl	0.139	0.052	0.158	0.067	-0.066	0.122	-0.149	-0.412	-0.051	-0.082	-0.345	0.093	0.062
HER-Trawl	0.011	0.012	-0.065	-0.305	0.048	0.068	0.032	-0.030	-0.076	-0.071	-0.014	-0.236	0.012
SPR-Trawl	-0.017	-0.284	0.046	0.024	0.049	-0.006	-0.020	-0.011	-0.007	0.006	-0.014	0.030	-0.254

Figure S7. Mixed Trophic Impact values for the year 2094 as produced by the model using the diet matrix as detailed in Table S4 (upper panel), and MTI values for the year 2094 if seal consumed exclusively herring, sprat and cod within the studied system (i.e. redistributing all the 'import' diet proportionally into the modelled prey, that is Import diet = 0).

Supplementary Bibliography:

Aydin, K. Y. 2004. Age structure or functional response? Reconciling the energetics of surplus production between single-species models and ECOSIM. *African Journal of Marine Science* 26. Taylor & Francis Group : 289–301. doi:10.2989/18142320409504062.

Chesson, J. 1983. The Estimation and Analysis of Preference and Its Relatioship to Foraging Models. *Ecology* 64. Wiley-Blackwell: 1297–1304. doi:10.2307/1937838.

Christensen, V., and C. J. Walters. 2004. Ecopath with Ecosim: Methods, capabilities and limitations. *Ecological Modelling* 172: 109–139. doi:10.1016/j.ecolmodel.2003.09.003.

Coll, M., A. Bundy, and L. J. Shannon. 2009. Ecosystem Modelling Using the Ecopath with Ecosim Approach. In *Computers in Fisheries Research*, 225–291. Dordrecht: Springer Netherlands. doi:10.1007/978-1-4020-8636-6_8.

Gales, N., M. Hindell, and R. Kirkwood. 2003. Marine Mammals: Fisheries, Tourism and Management Issues: Fisheries, Tourism and Management Issues.

Hammill, M. O., and G. B. Stenson. 2000. Estimated Prey Consumption by Harp seals (Phoca groenlandica), Hooded seals (Cystophora cristata), Grey seals (Halichoerus grypus) and Harbour seals (Phoca vitulina) in Atlantic Canada. *J Northwest Atl Fish Sci* 26. doi:10.2960/J.v26.a1.

Hanson, N., E. L. Jones, and R. N. Harris. 2017. Multi-decadal and ontogenetic trophic shifts inferred from stable isotope ratios of pinniped teeth. *Oikos*. doi:10.1111/oik.04441.

Hårding, K. C., T. Härkönen, B. Helander, and O. Karlsson. 2013. Status of Baltic grey seals: Population assessment and extinction risk. *NAMMCO Scientific Publications* 6: 33–56. doi:10.7557/3.2720.

Hårding, K. C., T. J. Härkönen, and O. Karlsson. 2015. *Suggestion for the development of an additional measure for the 'Nutritional Status of Seals' indicator. Core indicators and other activities under the Seal Expert Group teams. HELCOM.* Berlin, Germany, 2-4 December 2015.

Harvey, C. J., S. P. Cox, T. E. Essington, S. Hansson, and J. F. Kitchell. 2003. An ecosystem model of food web and fisheries interactions in the Baltic Sea. *ICES Journal of Marine Science* 60: 939–950.

Hiby, L., T. Lundberg, O. Karlsson, J. Watkins, M. Jüssi, I. Jüssi, and B. Helander. 2007. Estimates of the size of the Baltic grey seal population based on photo-identification data. *NAMMCO scientific publications* 6: 163–175.

Innes, S., D. M. Lavigne, W. M. Earle, and K. M. Kovacs. 1987. Feeding Rates of Seals and Whales. *J Animal Ecol* 56: 115. doi:10.2307/4803.

Link, J. S. 2010. Adding rigor to ecological network models by evaluating a set of pre-balance diagnostics: A plea for PREBAL 221: 1580–1591. doi:10.1016/j.ecolmodel.2010.03.012.

Lundström, K. 2012. Assessment of dietary patterns and prey consumption of marine mammals: Grey seals (Halichoerus grypus) in the Baltic Sea. Gothenburg: Swedish University of Agricultural Sciences (SLU) - University of Gothenburg.

Lundström, K., O. Hjerne, K. Alexandersson, and O. Karlsson. 2007. Estimation of grey seal (Halichoerus grypus) diet com- position in the Baltic Sea. *NAMMCO Sci. Publ* 6: 177–196.

Lundström, K., O. Hjerne, S.-G. Lunneryd, and O. Karlsson. 2010. Understanding the diet composition of marine mammals: grey seals (Halichoerus grypus) in the Baltic Sea. *ICES Journal of Marine Science* 67: 1230–1239. doi:10.1093/icesjms/fsq022.

Mackinson, S., M. Vasconcellos, T. Pitcher, C. Walters, and K. Sloman. 1997. Ecosystem impacts of harvesting small pelagic fish in upwelling systems: using a dynamic mass-balance model. In *Forage fishes in marine ecosystems*, 1:731–748.

Mark Jessopp, M. Cronin, T. Hart, M. Jessopp, M. Cronin, and T. Hart. 2013. Habitat-Mediated Dive Behavior in Free-Ranging Grey Seals. *Plos One* 8. Public Library of Science: e63720.

Murie, D. J., and D. M. Lavigne. 1992. Growth and feeding habits of grey seals (Halichoerus grypus) in the northwestern Gulf of St. Lawrence, Canada. *Canadian Journal of Zoology* 70. NRC Research Press: 1604–1613. doi:10.1139/z92-221.

Niiranen, S., T. Blenckner, O. Hjerne, and M. T. Tomczak. 2012. Uncertainties in a Baltic Sea Food-Web Model Reveal Challenges for Future Projections. *AMBIO* 41. Springer Netherlands: 613–625. doi:10.1007/s13280-012-0324-z.

Perrin, W. F., B. G. Würsig, and J. G. M. Thewissen. 2009. *Encyclopedia of marine mammals*. Elsevier/Academic Press.

Plagányi, É. E., and D. S. Butterworth. 2004. A critical look at the potential of Ecopath with ecosim to assist in practical fisheries management. *African Journal of Marine Science* 26: 261–287. doi:10.2989/18142320409504061.

Tomczak, M. T., S. Niiranen, O. Hjerne, and T. Blenckner. 2012. Ecosystem flow dynamics in the Baltic Proper—Using a multi-trophic dataset as a basis for food–web modelling. *Ecol Model* 230: 123–147. doi:10.1016/j.ecolmodel.2011.12.014.

Tomczak, M. T., J. J. Heymans, J. Yletyinen, S. Niiranen, S. A. Otto, and T. Blenckner. 2013. Ecological Network Indicators of Ecosystem Status and Change in the Baltic Sea. *PLoS ONE* 8: 1–11. doi:10.1371/journal.pone.0075439.

Ulanowicz, R. E., and C. J. Puccia. 1990. Mixed Trophic Impacts in Ecosystems. *Coenoses* 5: 7–16.

Walters, C., V. Christensen, and D. Pauly. 1997. Structuring dynamic models of exploited ecosystems from trophic mass-balance assessments. *Reviews in Fish Biology and Fisheries* 7. Kluwer Academic Publishers: 139–172. doi:10.1023/A:1018479526149.

Walters, C., V. Christensen, W. Walters, and K. Rose. 2010. Representation of Multistanza Life Histories in Ecospace Models for Spatial Organization. *Bulletin of Marine Science* 86: 439–459. doi:papers3://publication/uuid/F835554D-9C95-4A26-AA0B-31166058CC5B.
